# Towards programmable friction: control of lubrication with ionic liquid mixtures by automated electrical regulation

**DOI:** 10.1038/s41598-020-74709-2

**Published:** 2020-10-19

**Authors:** Felix Gatti, Tobias Amann, Andreas Kailer, Norman Baltes, Jürgen Rühe, Peter Gumbsch

**Affiliations:** 1 MicroTribology Center μTC, Fraunhofer Institute for Mechanics of Materials IWM, Woehlerstraße 11, 79108 Freiburg, Germany; 2 Fraunhofer Institute for Chemical Technology ICT, Joseph-von-Fraunhofer-Str. 7, 76327 Pfinztal, Germany; 3 IMTEK-Department of Microsystems Engineering, University Freiburg, Georges-Koehler-Allee 103, 79110 Freiburg, Germany; 4 Institute for Applied Materials-Computational Materials Science IAM-CMS, Karlsruhe Institute of Technology, Straße am Forum 7, 76131 Karlsruhe, Germany

**Keywords:** Chemistry, Engineering, Materials science

## Abstract

For mechanical systems in relative motion it would be fascinating if a non-mechanical stimulus could be used to directly control friction conditions. Therefore, different combinations of lubricants and external triggers for tribological influence have already been investigated. We show that when two metallic friction partners are lubricated with ionic liquid mixtures (ILM), consisting of long-chain cation and two different high charge/mass ratio anion containing ILs, the application of an electric impulse induces a permanent change of the frictional response. Such mixtures are able to alter the coefficient of friction (COF) to a greater extent, more accurately and faster than the respective single-component ILs. This change in the frictional properties is presumably due to changes in the externally induced electrical polarization at the surface, which influences the molecular adsorption, the exchange of adsorbed ions and their molecular orientation. The correlation between surface charges and friction can be used to control friction. This is achieved by implementing an electric tribo-controller which can adjust preset friction values over time. Programming friction in this way is a first step towards tribosystems that automatically adapt to changing conditions.

## Introduction

The vision underlying the development of programmable materials is to integrate functions directly into the material that can be modified or controlled. Control can for instance be exerted via an external electrical field. This external trigger could be an impulse to bring about a permanent change in the system. So far, the implementation of complex functions has usually been carried out via separate sensors and actuators. Function integration into the material itself constitutes a paradigm shift in the design process. Programmable materials and components made of these can directly be controlled and thereby replace complex multicomponent systems. This will enable programmable materials not only to react to direct user intervention, but also to adapt automatically to changing conditions.

A particularly large potential for the technical application of programmable materials arises in the field of tribological optimization and friction control. Friction occurs in all moving components and causes enormous ecological costs^1^. Correspondingly, in situ control of friction^2,3^, adaptive friction change^4^ and superlubricity^5–7^ were identified as the greatest challenges in tribology. Switchable or controllable friction is thereby recognized as an increasingly significant and important topic^8–11^.

The traditional approach to optimize friction and wear is to adapt the material and lubricant to the respective application or tribological system. Since the coefficient of friction (COF) is a complex function of several parameters, the system cannot be optimized for all operating conditions. This problem occurs in particular when the load spectrum (e.g. forces, speeds, temperatures) changes during operation or when, as in an automobile, several tribological contacts are lubricated with only one lubricant. In contrast, programmable materials shall allow programming of a COF into a technical application in order to stabilize a best possible operating point dynamically depending on the prevailing load collective.

**Figure 1:**
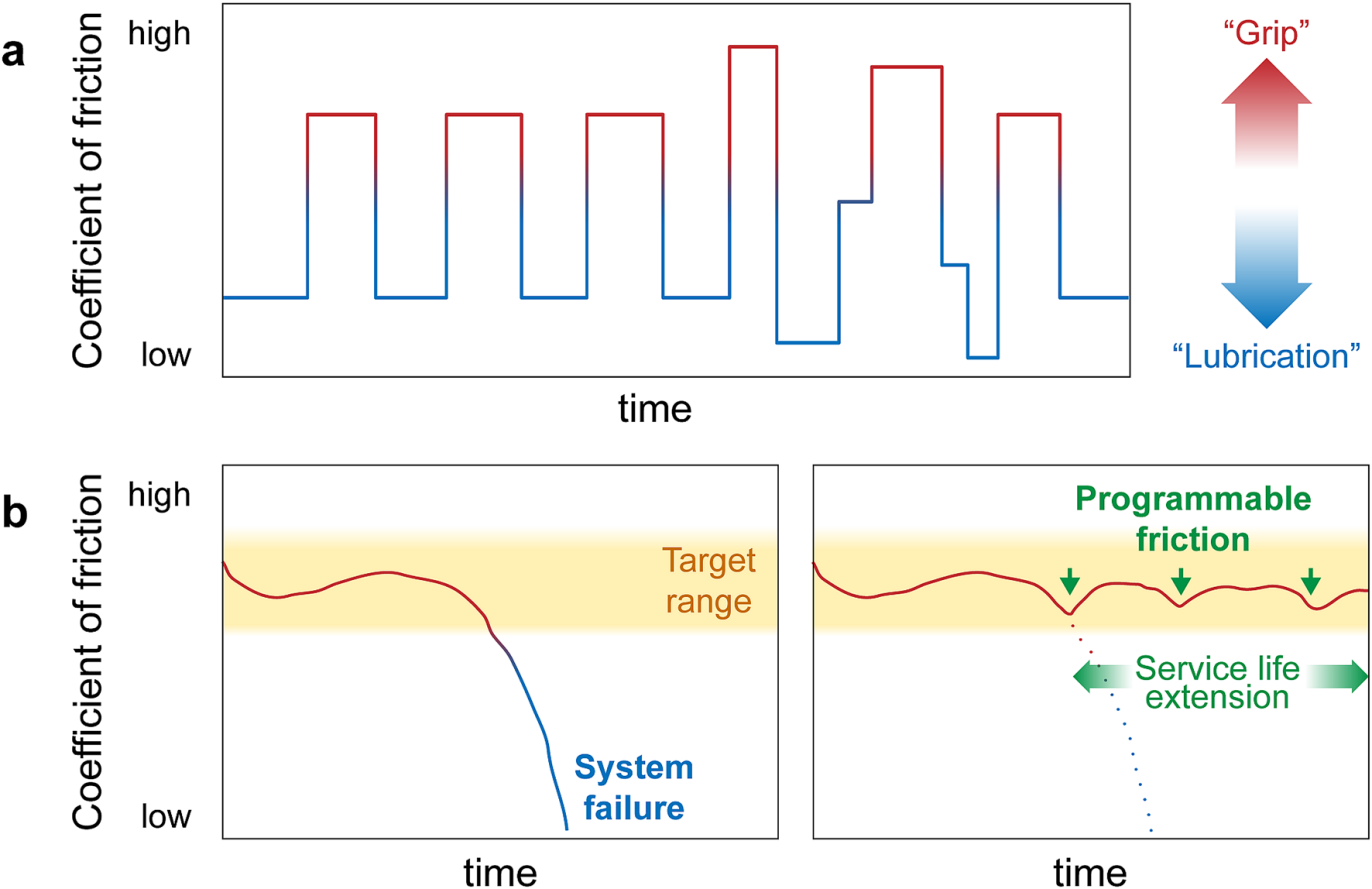
Motivation and aim of the project: (**a**) The vision of this work is to program friction systems in such a way that they are always running in the optimum operating state over the entire application parameters. Basically, high ("grip") or low ("lubrication") COFs or a variation of both ("positioning") can be desired. By programming COFs at certain test times, this work lays the scientific basis for the development of such programmable friction systems. (**b**) Typical course of the COF of a clutch lining during a life cycle with the target area colored yellow. The COF of a clutch must remain stable in a certain range to ensure power transmission. If the COF falls below this range, the system will fail (**b** left). In the event of a system failure, an external applied electrochemical potential could lead to an increase in the COF, so that the system failure does not occur and the service life of the clutch is considerably extended (**b** right).

Since friction can be both beneficial and detrimental, it is identified that its control is the key (Fig. 1a) to increase system efficiency, load capacity and durability^12^. The advantage of low friction in plain and roller bearings in respect to energy consumption or longevity is obvious. The importance of high friction in clutch transmission is exemplarily shown in Fig. 1b which sketches a typical COF curve of a clutch lining in the course of its life cycle. A high COF is required to ensure that the power transmission in the clutch takes place with minimum energy loss and without shuddering. As soon as the COF falls below the target range (yellow-colored regime in Fig. 1b), the system can no longer transmit the necessary forces and fails. An external potential resulting in an increase in the COF could increase the time of system failure and thus the service life of the clutch and be used to give an early warning of the end of life. While principally feasible, investigations on switchable or adjustable friction were hitherto mainly demonstrated for low contact pressure, low shear rate and with atomically smooth surfaces^13^. The challenge is to transfer and utilize the effects that were observed on a nanoscale to technologically relevant macroscopic systems^14^.

Achieving controllable lubrication, where friction occurs or nearly disappears as needed, has the potential to improve the performance of tribological systems^15^. Ionic liquids (ILs) have been identified as a new class of lubricants whose specific friction properties depend on their chemical structure^16,17^. On the nanoscale, friction is determined by the formation of an adsorbed near-surface structure of molecules of the IL^18^. Confinement can cause a phase change to a solid like behavior of adsorbed layers of IL^19^. Calculations of the interface structures of IL between gold electrodes showed that electrification leads to a layering of anions and cations at negative and positive surface charge densities of more than 20 μC/cm^2^^20^. These interactions are intensified on charged surfaces and lead to reformation of a strong interface layer^21–23^. Modifying the electric potential results in a change of the fluid-surface interactions and therefore the COF^24–26^ and may even reach superlubricity^27^. Preliminary work with ILs revealed that macroscopic friction and wear behavior can be improved by electrically^28,29^ and galvanically^30^ induced potentials. Mixing two ILs allows a precise adjustment of their dielectric properties^31^.

The aim of this work is to implement a system where the COF can be controlled in a defined and reliable way, which even allows programming a tribological system to control itself and maintain given friction values e.g. as a function of time (COF = f(t), Fig. 1a). The three major challenges are: (I) Control: Electrochemically influence surface interactions to control the tribological behavior by an external trigger and understanding the dependencies; (II) Programming: Tribosystem adjusts itself to programmed friction values; (III) Implementation: Transfer to a technical application.

The main idea followed here is to lubricate a tribological system with a mixture of ionic liquids (ILM) for which the polarization at charged tribological surfaces should lead to different arrangements of the different ions in the ILM. It is demonstrated here that control (I) of the COF can be achieved through its dependency on surface charges. An external electrical control can then be used to program (II) friction in a macroscopic tribological system that can principally be transferred to a technical application like a clutch (III).

## Results

**Figure 2:**
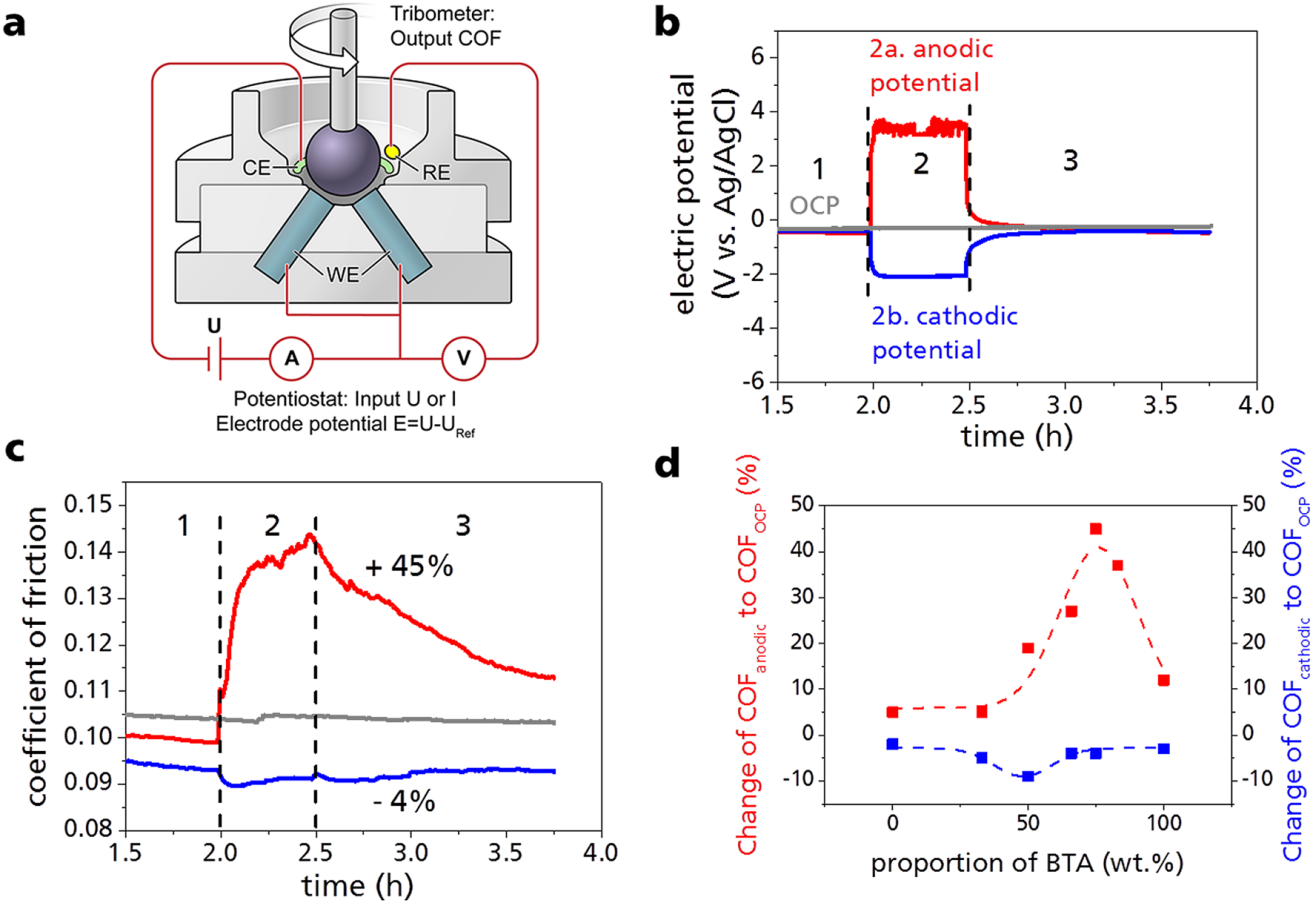
(**a**) Rotating ball-on-3-pin friction geometry in an electric insulated measuring cell coupled with a potentiostat to apply electric potentials. The lower pins are set as working electrode (WE), platinum is used as counter electrode (CE) and Ag/AgCl electrode as reference electrode (RE). Test parameter were set to 50 N, 100 rpm (0.05 m/s) and room temperature (25 °C). To analyze the influence of different electrochemical potentials, tribological tests were performed at open circuit potential (OCP), cathodic and anodic polarization. (**b**) The test is divided into three phases: (1) running-in without external potential at OCP (2 h, not fully shown); (2) anodic or cathodic surface polarization (0.5 h); (3) running-out at OCP (1.25 h). The tests were performed with the pure ionic liquids [P66614][DEHP] (D) and [P66614][BTA] (B) and mixtures of both in different weight ratios: D2:B1 (two parts [P66614][DEHP] and one part [P66614][BTA]), D1:B1, D1:B2, D1:B3 (shown in **c**), D1:B5. (**c**) Here is an example of the resulting change in the COF shown for the mixture D1:B3. If an anodic potential (red line) is applied, the COF increases by 45%. Under cathodic loading (blue line) the COF decreases by 4%. (**d**) The mixing ratio of the ILs influences the change of COF at anodic (red spots) and cathodic (blue spots) surface polarization compared to COF without external potential. The maximum range of switchability of COF is achieved by anodic and cathodic polarization for different mixtures.

The experimental setup consists of a tribological cell which is connected to a potentiostat to induce electrochemical surface potentials in the sliding contacts between the rotating ball and the three lower stationary pin, which are set as the electrochemical working electrodes (WE). (Fig. 2a). The controllability of the COF was first tested with a constant current between WE and counter electrode (CE), which results in either anodic or cathodic polarization on the charged surface. Two different ILs and their mixtures were used. The tribological tests were performed at a normal force of 50 N (23.6 N per pin) on a ½ inch 100Cr6 steel ball rotating to induce sliding contacts on three 100Cr6 steel pins at 100 rpm (speed: 0.05 m/s) at room temperature (25 °C) for 3.75 h. Two commercially acquired ionic liquids with the same phosphonium cation [P66614] were used. The anions were either a phosphate-based variant [DEHP] and a sulfonyl imide variant [BTA]. The tests were carried out using pure [P66614][DEHP] (abbreviated as D), pure [P66614][BTA] (abbreviated as B) and mixtures of both in different proportions. The selection of these two ionic liquids was based on previous experience of their respectability to friction modification^29^. The specifics are long alkyl chains in the cation to give non-polar parts in the polar structure of two charged ions, which in a future step ensures at least a small miscibility with a non-polar oil^30^. It has been reported that it is mainly the anion that interacts with the surface^32^. Therefore, different anions were selected to study their interaction with the electrically charged surface.

To avoid running-in effects and to study system behavior, the experiments were started with an initial step with constant tribological parameters for two hours without external potential (open circuit potential, OCP), then a constant current of ± 300 µA was set for 0.5 h and afterwards the experiment continued at OCP for 1.25 h. For brevity we speak about an anodic current (+ 300 µA) if the current creates an anodic charged surface and about cathodic current (− 300 µA) for the reverse situation.

Figure 2b,c shows the results of the tribological test using the ionic liquid mixture consisting of one part [P66614][DEHP] and three parts [P66614][BTA] (abbreviated D1:B3). The use of an anodic current leads to an electric potential which is in the range of + 4 V versus the reference electrode Ag/AgCl with a scatter of about ± 500 mV (Fig. 2b). The cathodic potential is in the range of − 2 V versus Ag/AgCl without any scatter. The relatively high voltages are due to the construction of the tribological cell, the distance between the working and counter electrodes and the area of these two electrodes. After the tribotest the same OCP is reached as before polarization. During the running-in phase (not completely shown in figures, Fig. S1), without external potential, but measuring the open circuit potential (OCP), a slight reduction of the COF occurs due to the roughness and chemical adaptation of the friction partners and the abrasion of the asperities.

When an anodic current is applied, the COF increases by 45% compared to the non-polarized state (Fig. 2c). In contrast, at cathodic current only slightly changes the COF by about 4%. It can be seen that there is a significant time lag between applying the current and reaching the maximum or minimum friction value, which indicates dynamic and continuous formation but also changes in the lubrication film in the friction gap. Tribological tests using pure [P66614][DEHP], pure [P66614][BTA] and mixtures of both ILs in the weight proportion of D1:B1, D2:B1, D1:B2 and D1:B5 were carried out with the same sequences as in Fig. 2b. The applied external electric potential, tribological characteristics and OCP curves during running-in are illustrated in the Supplementary Material (Figs. S1, S2). Figure 2d summarizes the maximum change in the COF of these fluids that can be achieved by charged surfaces. A larger proportion of [P66614][DEHP] quickly reduces the influence on the COF. For all fluids there is the same tendency to lower COF at cathodic and higher COF at anodic potential. The results show that the influence of polarization of the ILs on the COF increases up to a certain amount of [P66614][BTA] in [P66614][DEHP] and then decreases again. The strongest change in the COF compared to the non-polarized state is achieved at a proportion of approximately 50% [P66614][BTA] when cathodic potentials are used. For the anodic potential the maximum is at a proportion of 75% [P66614][BTA].

Wear scars, which were measured with a 3D laser microscope, are visible on the pins in the sliding direction (Fig. S3). IL-mixtures cause less wear than pure ILs, however anodic surface charges and a higher proportion of [P66614][BTA] leads to an increase of wear. No chemical changes of the ILs were detected after tribotest at anodic and cathodic potential using IR-spectroscopy (Fig. S4).

These experiments show that the sensitivity and quantity of switching and wear correlates with the mixing ratio of the two ILs. D1:B3 with maximum COF switching capacity was identified as the most suitable ILM. These results form the basis for automatic voltage regulation to achieve a preset COF over time.

**Figure 3:**
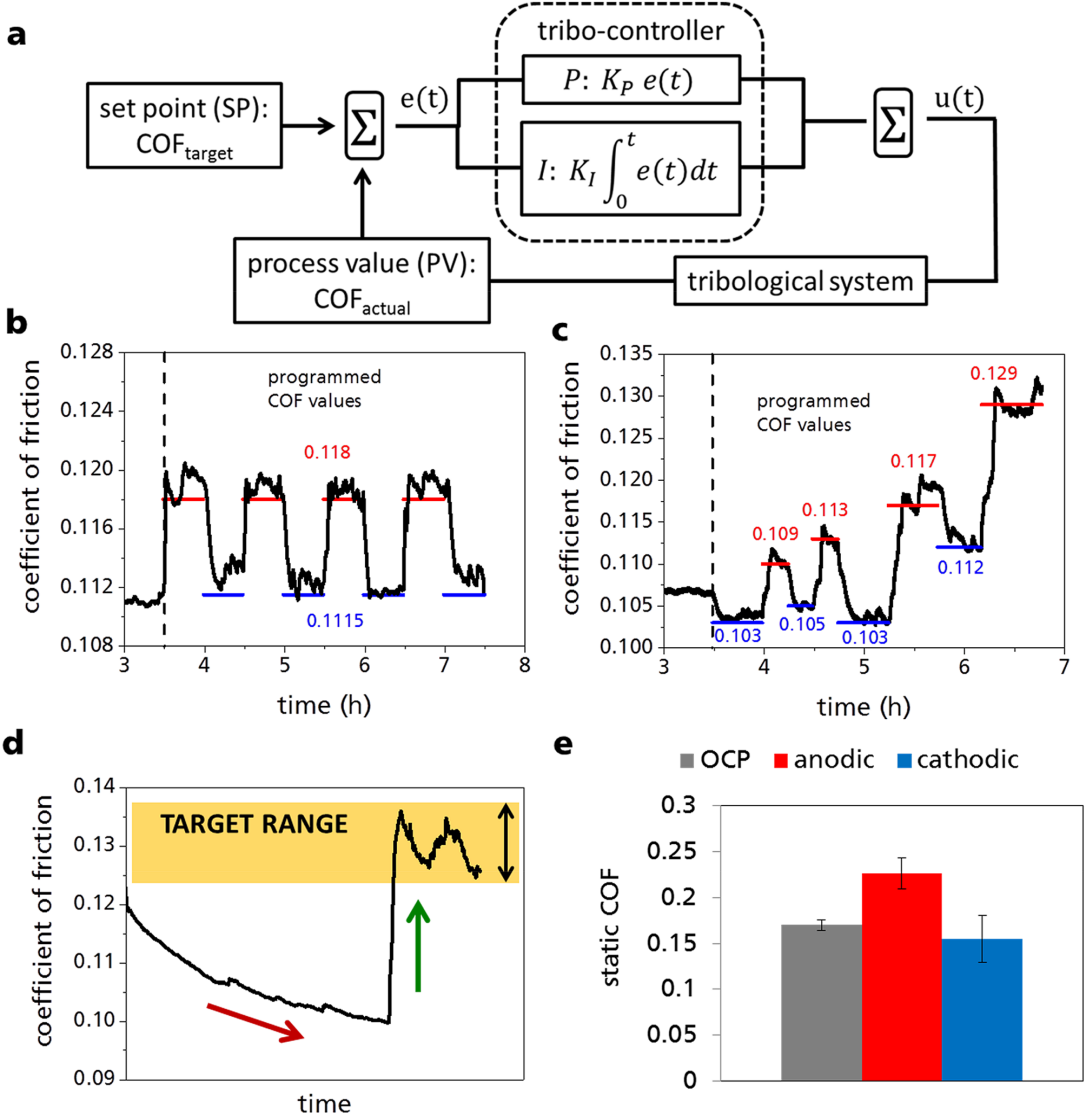
(**a**) Functionality of the tribo-controller: The time-dependent deviation e(t) is calculated from the difference between the current COF_actual_ (SP) and the preset COF_target_ (PV). Then the actuating variable u(t) is calculated via a proportional P and an integral I component. In these experiments the manipulated variable is the current, which is controlled by the potentiostat. (**b**,**c**) The tribo-controller processes the input values (programmed COF at certain test times) and controls the output (electric current). After running-in phase without external potential (OCP, not shown) programmed COFs (red and blue line) were automatically adjusted by the tribo-controller by regulating the electrical current: (**b**) multiple adjustment of the same COF 0.118 and 0.1115. (**c**) Multiple adjustment of different torques in the range of 0.103–0.129. These tribological tests with the tribo-controller were conducted using the ionic liquid mixture D1:B3 (one weight part [P66614][DEHP] and three weight parts [P66614][BTA]). (**d**) Tribological test in which it could be shown that with the aid of an electrical potential the COF could be controlled and maintained in a target range after a certain running-in period. (**e**) The results of the static friction tests with anodic (red) and cathodic (blue) polarization of the pins are shown. To determine the static friction, the torque of the ball is increased until the ball breaks away from the pins. The moment of breakaway describes the static friction of the tribological system.

The finding that COF correlates with the surface charge and the ILM used allows the COF to be specifically controlled by external electrical voltages. This correlation leads to the concept of the tribo-controller, which is shown schematically in Fig. 3a. The tribo-controller receives as input both the current process value PV of the COF due to the coupling with the tribological system and the set point SP value of the COF. The difference between PV and the SP is minimized using a proportional component P and an integral component I of an electric control unit acting on the electric current in the tribological cell. The tribo-controller operates with a frequency of 1 Hz and current limits of ± 500µA. Values of the control parameters must be adapted to the respective tribological system. The terms of Fig. 3a are explained in detail in the part Materials and Methods in the supplementary material.

Since the ILM D1:B3 leads to the strongest change in COF by anodic polarization this lubricant was used for further friction tests with the tribo-controller. Figure 3b,c shows the programmability of the COF. Preset friction values (SP) at certain times were realized. Figure 3b illustrates that the programmed COFs of 0.1115 and 0.1180 for predefined test times can be achieved reversibly. Increase of the COF is realized by anodic and reduction of the COF by cathodic polarization of the ILM. Multiple programming of different COFs over a wide range from 0.103 to 0.129 (± 25%,) is shown in Fig. 3c. In these experiments, the main focus was on the accuracy of the tribo-controller with respect to the preset COFs and not on exhausting the maximum switchability of the IL-mixture. The course of the required current during the two experiments and the area under the curve for each COF change (electric charge quantity, ECQ) are shown in the supplementary material (Fig. S5). In both tests the ECQ for the entire switching operation is between 0.03 and 0.60 A·s. At the beginning of the anodic switching process, the current flow increases and the COF becomes higher. Almost no current is required to maintain the preset COF after a few minutes. In contrast, cathodic switching operations, decreasing the COF, sometimes require significant currents for the entire ½ h of low COF-operation. Smaller currents (maximum + 200 µA) are needed to increase the COF than to decrease the COF (maximum − 500 µA). The ECQ for reducing the COF at cathodic potential does not seem to be a deterministic value but seems to depend on the sample history. At anodic switching processes it can be seen that only a pulse of current or potential is sufficient to bring about the necessary change in the system. The change in COF can then be maintained without further current flow (Fig. S6).

The implementation of programmable friction in a friction system mimicking a clutch is shown in Fig. 3d. With this ILM and the setup of a tribo-controller with a tribometer it is shown that the COF after a certain period can be brought to a target range and kept there so that power transmission is always guaranteed.

Static friction values can also be influenced using electric potentials with the ILM D1:B3 (Fig. 3e). In order to determine the static friction, a test was carried out in the same tribological system and in the same setup, in which the torque of the ball was increased until the onset of relative motion between ball and pins. The threshold value for the onset of sliding describes the static friction of this tribological system. This test was performed at + 300 µA as well as at − 300 µA. For the reference measurement the static friction was also measured without any current (OCP). By applying an anodic potential, the static COF can be increased by 30% compared to the non-polarized state. Cathodic polarization leads to a reduction of 10%. These results prove that the COF cannot only be controlled in dynamic systems, but also the static friction can be influenced.

## Discussion

The difference in anodic and cathodic response indicates that the anions have a stronger and faster interaction with the charged surface than the cations. This hypothesis is supported by the fact that in anodic switching processes only a pulse of current is necessary to achieve the change in COF. The system can maintain the changed friction state without further current flow. To release the smaller anions from the surface, where the charge shielding is lower, a higher charge density is required and the entire process depends on the previous anodic charging of the surface.

**Figure 4:**
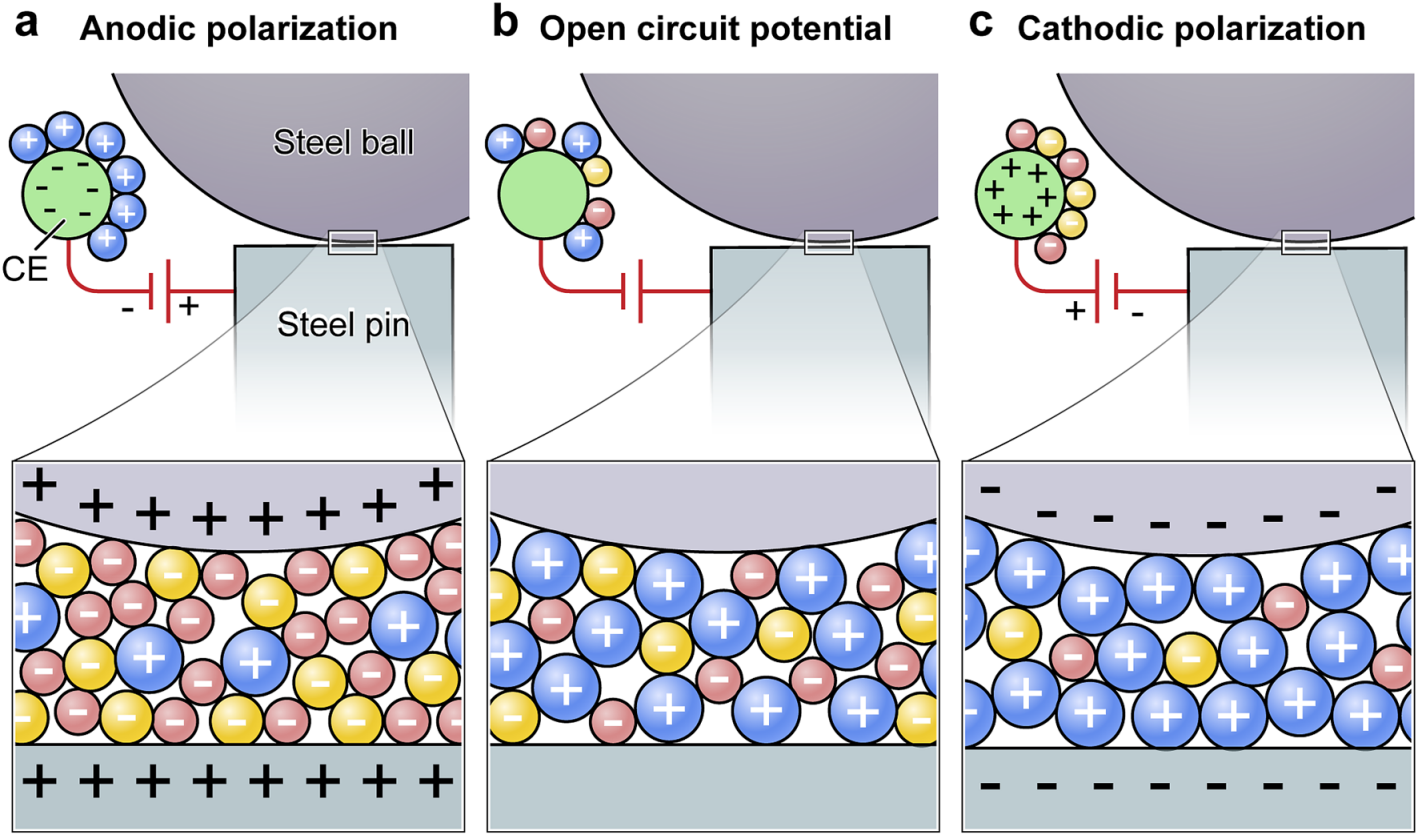
Proposed model for the molecular layering of an ionic liquid between two metal surfaces (represented in blue: the cation [P66614]; in red: the anion [BTA] and in yellow the anion [DEHP]). (**a**) Anodic polarized surface with near surface layering of small anions. The anodic polarization forces the cations with their oil-like side groups out of the friction gap and the friction increases. (**b**) Non-polarized state of the ionic liquid between the two surfaces. (**c**) Cathodic polarized surface with near surface layering of the large cations. Due to the size and the oil-like side chains of the cations, a mechanically stable lubricating film is formed, which reduces friction.

Figure 4 illustrates a model of the interactions of the ionic liquids with the surface. According to the findings of Gil et al.^33^ using molecular dynamics simulations of ILs at charged surfaces, it is assumed that the near-surface structure of ILs is altered by the external polarization. In addition with our finding that the externally induced potential on the pins causes the same charge on the rotating ball (Fig. S7) this model is extended here. During the running-in phase at OCP the topologies of the two surfaces adapt to each other through wear and generates reasonably flat load-bearing terraces on both surfaces. It can be assumed that the anions and cations of the ionic liquid are statistically distributed in the lubricant, since the existing charge of the steel surfaces during OCP is small in comparison to the polarizations (Fig. 4b). Surface charges change this distribution and more equally charged ions are deposited on the surface. Anionic polarization induces an increase in the concentration of the smaller anions in the near-surface contact (Fig. 4a). This formation of a thin anion layer causes an increase of cation concentration in the bulk and on the counter electrode. In contrast, cations are enriched on the surface during cathodic polarization and the steel pin and ball are cathodically protected (Fig. 4c). Since the cations have a larger volume than the anions and have lubricating properties due to the long alkyl chains, this reduces wear and friction.

In order to describe the influence of potential-related layer formation on the change in COF, the layer thickness and the friction regime (lambda value) were calculated according to Popov^34^. These values are correlated with the COF changes (Fig. S8). Due to wear, the initial contact pressure of 1150 MPa per pin decreases to about 200–400 MPa (Table S1). A lambda value (the lubricant film thickness (h) within the contact is divided by the composite roughness (σ) of the two surfaces forming the contact) of < 1 indicates that all tests are performed in the boundary lubrication regime. There is a tendency that in anodic switching, the smaller the lubricant film thickness and also lambda, the greater the change in the COF. At lower lubricant film thickness, the targeted electrochemical adsorption of the IL molecular layers has a greater effect on the coefficient of friction than at lower pressure and high lubricant film thickness. To check this, measurements were carried out for D1:B3 at normal loads of 15 N and 30 N (Fig. S9). Due to the lower normal force, higher film thickness and higher lambda values > 1 were formed at the same viscosity. The changes in the COF were 19% (15 N) and 25% (30 N) respectively compared to the 45% at 50 N. Due to the tribometer used, no normal forces higher than 50 N could be tested.

To what extent the switching capacity depends on the amount of current an experiment with D1:B3 at a current flow of 150 µA and 50 N was carried out (Fig. S10). At a lower anodic current pulse a smaller change in the coefficient of friction was induced (8%). It is assumed that the magnitude of the current pulse influences the thickness of the adsorbed molecular layers. In the IL-mixture used, the anions have a smaller molecular volume than the cations. This allows them to adsorb more effectively and faster on the surface (lower diffusion barrier, lower charge shielding, higher polarity). The initial current pulse is sufficient to induce a molecular order and maintain it without further current flow. The larger cations (charge shielding, steric hindrance) are not adsorbed on the surface with sufficient stability by a current pulse, so that it is necessary to maintain the current flow to keep the molecular layers stable and to induce a change in the coefficient of friction. Tests with higher currents did not lead to an increase in the change in COF compared to the tests with 300 µA. It can be assumed that with these parameters the maximum layer formation was achieved and additional layers are destroyed by the shear in the system at higher currents.

As shown in Fig. 2d, there is a small change of the COF in the pure ILs. The controllability of the tribological properties can however be increased by mixing two anions. The detailed reason for the different quantity of circuitry at different mixing ratios must be examined more closely in subsequent investigations. It can be assumed that the interactions described above depend on additional material-specific parameters like the molar volume, Coulomb interaction, dissociation energy of the individual ions and packing density. They determine the layering mechanisms which in turn change the tribological behavior.

The applied bias voltage on the substrates also leads to a change in surface energy. Since the surface energy of the substrate and the surface tension of the lubricant influence the adhesion of the ions and thus the lubricating properties, the interfacial parameters of the system must be taken into account^35–37^. [P66614][BTA] (γ: 27.4 mN/m, 0.59 ratio polar/dispersive) has nearly the same surface tension but higher polarity than [P66614][DEHP] (γ: 30.5 mN/m, 0.19 ratio polar/dispersive). This causes higher interfacial tension between steel and [P66614][BTA] (2.4–0.6 mN/m, Fig. S11). On the other side [P66614][BTA] has a higher spreading parameter (SP: 9.4–8.1 mN/m) which indicates better wetting on steel surface.

## Conclusion

IL-mixtures in which the cation contains long alkyl chains and the anion has a high charge/mass ratio can be used very advantageously as lubricants, and the COF can be changed by applying a single electrical anodic pulse. This behavior was used to set predetermined COF at certain points in time with the implementation of a PID controller. The reversible switchability of the COF was evaluated based on the friction regime and the control signal (electric current). The wear behavior, sensitivity and the extent of the switchability of the COF are influenced by the composition of the ILM and the electrical signal. Applying anodic potential, an initial current pulse is sufficient to change the COF. Without further current flow, a new stable friction state is formed. In contrast, upon application of a cathodic potential a continuous current flow is required to control the friction.

When the applied load/contact pressure and thus the lubricant film thickness in the friction gap is varied the extent of switching of the COF is changing accordingly. Lowering of the lubricant film thickness leads to an increase in the response of the friction force as a function of the applied potential. Potentially a higher film thickness decreases the influence of the surface polarization on the overall order in the friction gap.

The system described here can now be extended to two scenarios: First, an electrical impulse can be used to program the friction under computer control. This will allow switching from a high to a low friction situation simply by applying an electrical potential. Another attractive scenario is the coupling of the electrical input signal to the friction output and thus the generation of a system that automatically adapts to changes in the friction behavior and thus enables an autonomous adaptation of the friction to changes in the environment without the need of user intervention.

## Methods

### Instrumentation

The macroscopic friction behavior was investigated using a modified ball-on-3-pin tribometer (Co. Anton Paar, MCR501, Fig. 2a). As upper rotating test specimen a steel ball (100Cr6, 1.3505, Grade 28, DIN5401, diameter: ½’’, 60–66 HRC, 740–900 HV10, *R*_*a*_ = 0.26 ± 0.06 µm, *R*_*z*_ = 0.57 ± 0.14 µm; see Fig. S12, Supplementary) was used. An electrically insulated measuring cell made of Polyether ether ketone (PEEK) was used as a holder for the stationary pins. The three pins (100Cr6, 750–850 HV10, *R*_*a*_ = 0.32 ± 0.05 µm, *R*_*z*_ = 2.47 ± 0.29 µm; see Fig. S12, Supplementary) are connected as working electrodes (WE). The total contact area of the pins with the IL (1.1 ml) is 84.8 mm^2^. A platinum wire (60 mm^2^) was used as counter electrode (CE) and an Ag/AgCl electrode as reference (RE, Warner Instruments). The electrochemical potential was adjusted by the specification of the electrical current of ± 300 µA via potentiostat (LPG 03-50, Co. Bank Electronic). The relatively high electrical voltage, which must be induced to reach ± 300 µA, is due to the design of the electrochemical friction measuring cell. Due to the relatively small area of the counter electrode (61 mm^2^) and the large distance between counter and working electrode (approx. 4 mm) in combination with the limited conductivity of the ionic liquid or the IL-mixtures, an electrical resistance in the range of approx. 6–13 kΩ is created. The first experiments on the controllability of COF with the sequence as shown in Fig. 2b and the results in Fig. 2d were carried out with this 3-electrode setup. Later in combination with the tribo-controller, due to the small difference in the resulting potential, the Ag/AgCl reference electrode was omitted and the platinum electrode was connected in a 2-electrode setup both as counter electrode and reference electrode. After the tests, wear was determined using a 3D laser microscope (Keyence, VK-9710K).

The static friction was also tested in the model construction. The moment on the ball was increased until the ball slides off the three pins. In order to determine a scatter of the experiment, this experiment was repeated 5 times. The tribotests were all performed at 50 N, 100 rpm and room temperature.

### Tribo-controller

The experiments, in which the COF was programmed as a function of time, were carried out using a PI-controller. The controller determines the difference between a preset set point SP and a process value PV according to Eq. (1) to obtain the time-dependent deviation e(t).

1 $$ e\left( t \right) = SP - PV $$

The actuating variable u(t) is composed of a sum of a proportional component P and an integral component I. Equation (2) shows the calculation of P with the control amplification K_P_. P is proportional to e(t) and indicates how quickly the controller reacts to changes in e(t).

2 $$ P: u \left( t \right) = K_{P} e\left( t \right) $$

With the integral part I, control fluctuations and disturbances in the system can be avoided. Equation (3) shows that I is proportional to the integral of e(t) by the factor K_I_.

3 $$ I: u \left( t \right) = K_{i} \mathop \smallint \limits_{0}^{t} e\left( t \right)dt $$

### Materials

The ILs used in this study were Trihexyltetradecylphosphonium-*bis*(trifluoromethylsulfonyl)-imide [P66614][BTA] (IN-0021, Co. Iolitec, *η*: 310 mPa·s at 25 °C, 2400 ppm water, *κ*: 6.63 mS/cm at 25 °C, *M*: 391.3 g/mol, thermal decomposition: 270 °C; chemical structure see Fig. S13, Supplementary) and Trihexyltetradecylphosphonium-*bis*(2-ethylhexyl)-phosphate [P66614][DEHP] (CS-0957, Co. Iolitec, *η*: 1160 mPa·s at 25 °C, 433 ppm water, *κ*: 0.002 mS/cm at 25 °C, *M*: 805.3 g/mol, thermal decomposition: 220 °C; chemical structure see Fig. S13, Supplementary). These two ILs were mixed in five different mass ratios of [DEHP] to [BTA]: 2:1 (D2:B1), 1:1 (D1:B1), 1:2 (D1:B2), 1:3 (D1:B3) and 1:5 (D1:B5). The thermogravimetric analysis (TGA, see Fig. S14, Supplementary) and rheological behavior of the ILs are illustrated in the Supplementary Fig. S15. In contrast to [P66614][BTA], [P66614][DEHP] shows a shear thinning behavior at low shear rates and higher viscosity. The ILs were stored in a glovebox under nitrogen atmosphere to keep the water content of the ILs constant.

### Electrochemistry

Corrosive properties (Linear sweep voltammetry LSV, scan rate: 0.1 mV/s, potential range: − 1 to + 1 V; see Fig. S16, Supplementary) and electrochemical stability (cyclic voltammetry CV, scan rate: 100 mV/s, electrochemical window: − 10 to + 10 V, 10 cycles; see Fig. S17, Supplementary) of the ILs were measured in contact with 100Cr6 (Table S2). The electrochemical equivalent of the used 100Cr6 pin is *k*: 2.85E−4 g/A·s, which in combination with the alloying elements results in an equivalent weight of *Q*: 26.88 g/mol.[P66614][DEHP] has a more negative corrosion potential (*E*_*Corr*_) and lower corrosion current density (*i*_*Corr*_) than [P66614][BTA], which causes a lower corrosion rate (*C*_*R*_). CV measurements prove that no decomposition (*i* < 1 mA/cm^2^) of the ILs takes place in the electrochemical window of ± 10 V vs. Ag/AgCl. However, it must be noted that some reversible oxidation and reduction reactions occur within this window.

### Calculation of the Lamda-values according to Popov^34^

Lamda-values are used to determine which friction regime a system is in. For this purpose, the lubricant film thickness *h*_*0*_ was calculated using Eq. (4).

4 $$ h_{0} = \sqrt {\frac{l \cdot \eta \cdot \nu }{p}} $$

The contact length $l$, the viscosity η, the speed of the rotating ball ν and the resulting contact pressure p are used. Then the composite roughness σ of the two friction partners was calculated using Eq. (5).

5 $$ \sigma = \sqrt {\left( {R_{a1}^{2} + R_{a2}^{2} } \right)} $$

The lambda-values could then be calculated using Eq. (6).

6 $$ \lambda = \frac{{h_{0} }}{\sigma } $$

### Contact angle

Surface energies of the two ILs and their dispersive (D) and polar (P) properties were measured using sessile drop method (contact angle system OCA20, Co. Data Physics, fluid volume: 1 µl, efflux rate: 1 µl/s). In combination with the measured surface tension using pendant drop method (efflux rate: 1 µl/s) the polar and dispersive components of the lubricants were calculated. According to Fowkes method, the interfacial tension is calculated based on the surface energy and the surface tension of the lubricant and their polar and dispersive components.

## Supplementary information

Supplementary file 1

## Data Availability

All data is available in the main text or the supplementary materials.
