# Supplementary material for: Towards programmable friction: control of lubrication with ionic liquid mixtures by automated electrical regulation
Source: Sci Rep. 2020 Oct 19;10:17634. doi: 10.1038/s41598-020-74709-2 (PMC7572367; doi:10.1038/s41598-020-74709-2)
Supplement: Supplementary file 1 [file 41598_2020_74709_MOESM1_ESM.docx]

Supplementary information

Towards programmable friction: control of lubrication with ionic liquid mixtures by automated electrical regulation

*Felix Gatti^1^, Tobias Amann^1^, Andreas Kailer^1^, Norman Baltes^2^, Jürgen Rühe^3^, and Peter Gumbsch^1,4^*

^1^ F. Gatti, Dr. T. Amann, Dr. A. Kailer

Fraunhofer Institute for Mechanics of Materials IWM, MicroTribology Center μTC, Woehlerstraße 11, 79108 Freiburg, Germany

^2^ Dr. N. Baltes

Fraunhofer Institute for Chemical Technology ICT, Joseph-von-Fraunhofer-Str. 7, 76327 Pfinztal, Germany

^3^ Prof. J. Rühe

University Freiburg, IMTEK - Department of Microsystems Engineering, Georges-Koehler-Allee 103, 79110 Freiburg, Germany

^4^ Prof. P. Gumbsch

Karlsruhe Institute of Technology, Institute for Applied Materials - Computational Materials Science IAM-CMS, Straße am Forum 7, 76131 Karlsruhe, Germany.

Correspondence and requests for materials should be addresses to T.A. (tobi-as.amann@iwm.fraunhofer.de)


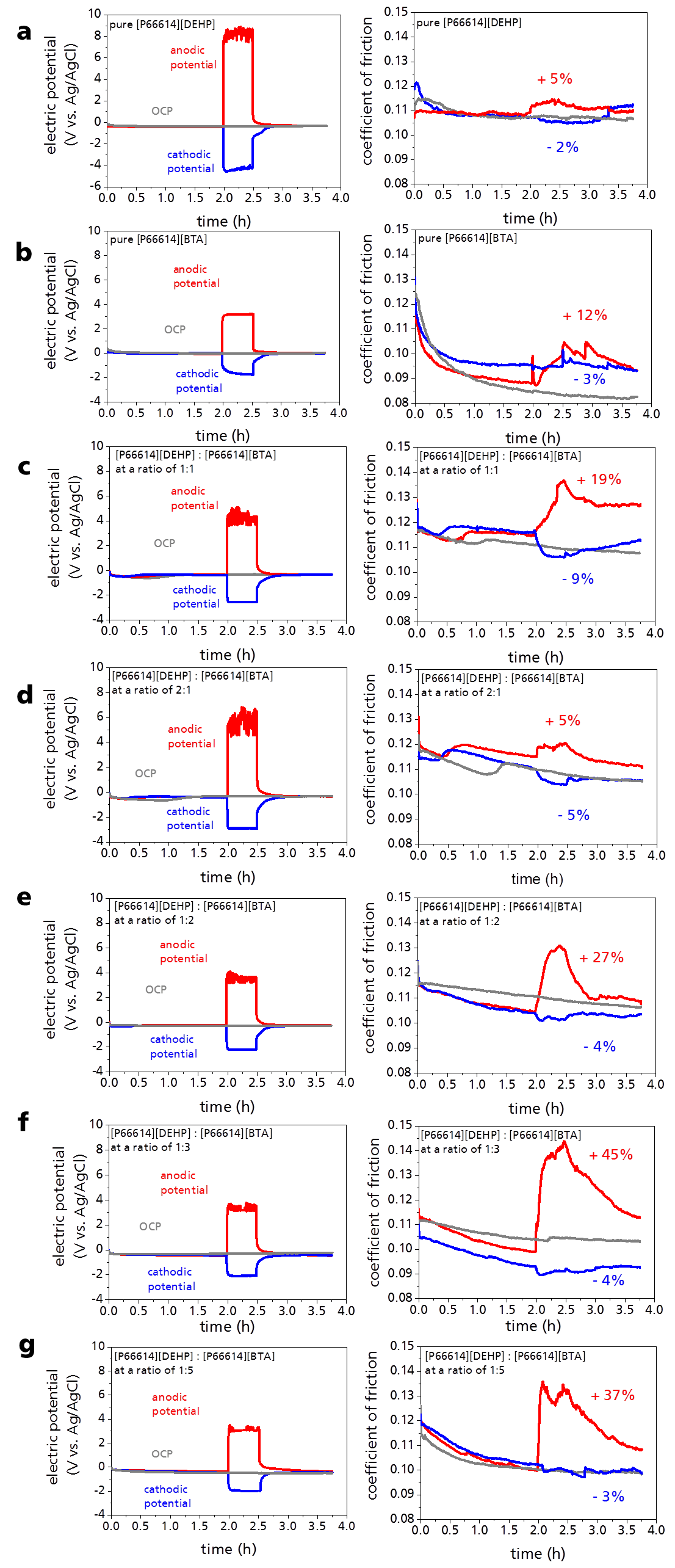


**Fig S1** Overview of the externally applied electrochemical polarization (left side) and the resulting effects on the tribological behavior (right side) of **a** pure [P66614][DEHP] and **b** pure [P66614][BTA], as well as the mixtures of [P66614][DEHP] (D) and [P66614][BTA] (B) in a weight proportion of **c** 1:1 (D1:B1), **d** 2:1 (D2:B1), **e** 1:2 (D1:B2), **f** 1:3 (D1:B5) and **g** 1:5 (D1:B5). The tribological test consists of a running-in phase of 2h at OCP, an anodic (red line, +300 µA) or cathodic (blue line, -300 µA) polarization of 0.5 h long and a running-out phase of 1.25h at OCP. The grey line describes the experiment with continuous OCP.


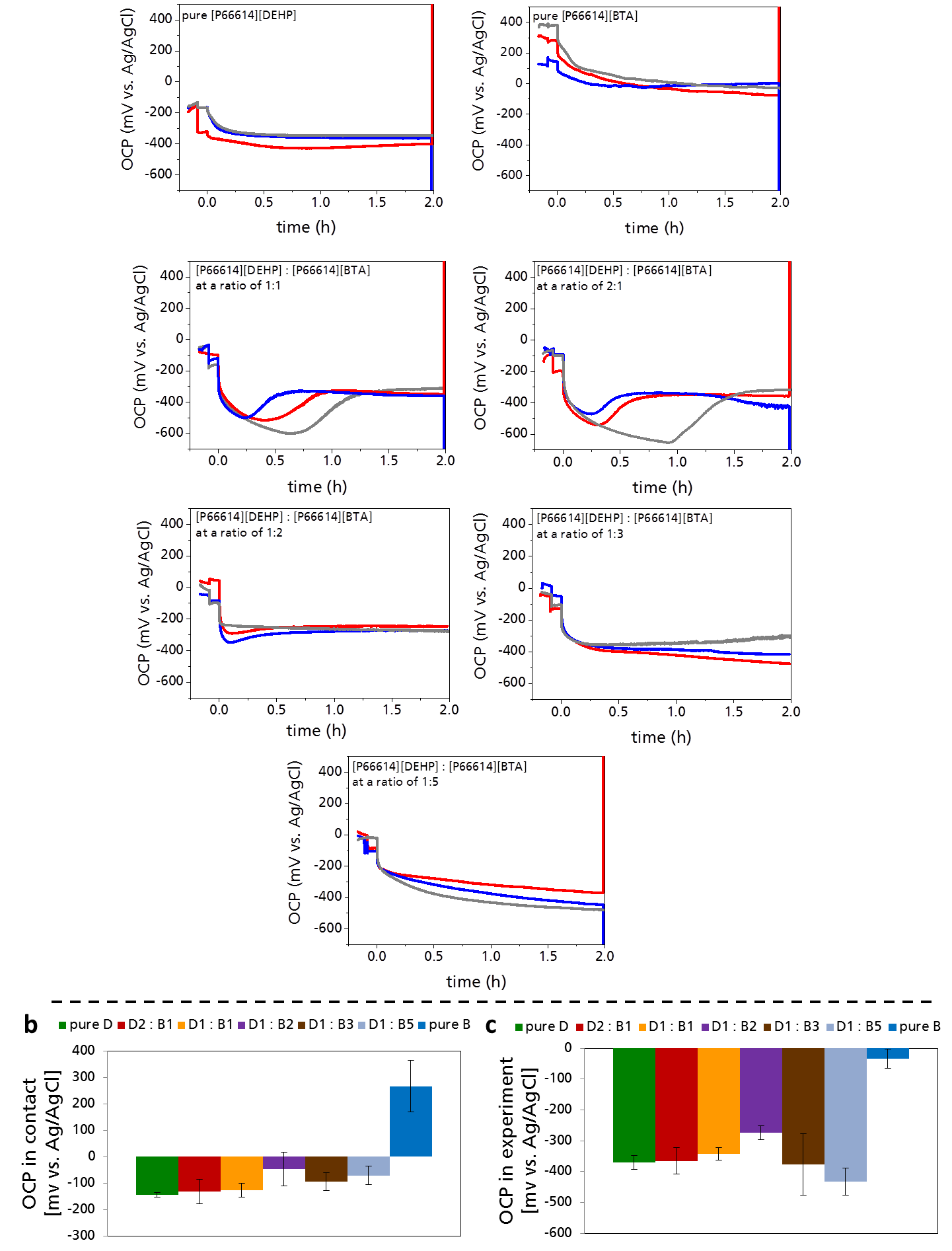


**Fig S2** **a** open circuit potential (OCP) of pure [P66614][DEHP], pure [P66614][BTA] and the mixtures of [P66614][DEHP] (D) and [P66614][BTA] (B) in weight ratios of 1:1 (D1:B1), 2:1 (D1:B2), 1:2 (D1:B2), 1:3 (D1:B3) and 1:5 (D1:B5) before the start of the friction test (5 min to 0 h) and during the running-in phase (to 2 h). After the running-in phase the experiments were then continued at OCP (grey line), cathodic (blue line) and anodic (red line) polarization for 30 minutes. **b** Values of OCP at contact of ball and pins just before the start of the friction test of all used lubricants. **c** Value of the OCP at the end of the running-in phase of all used lubricants.


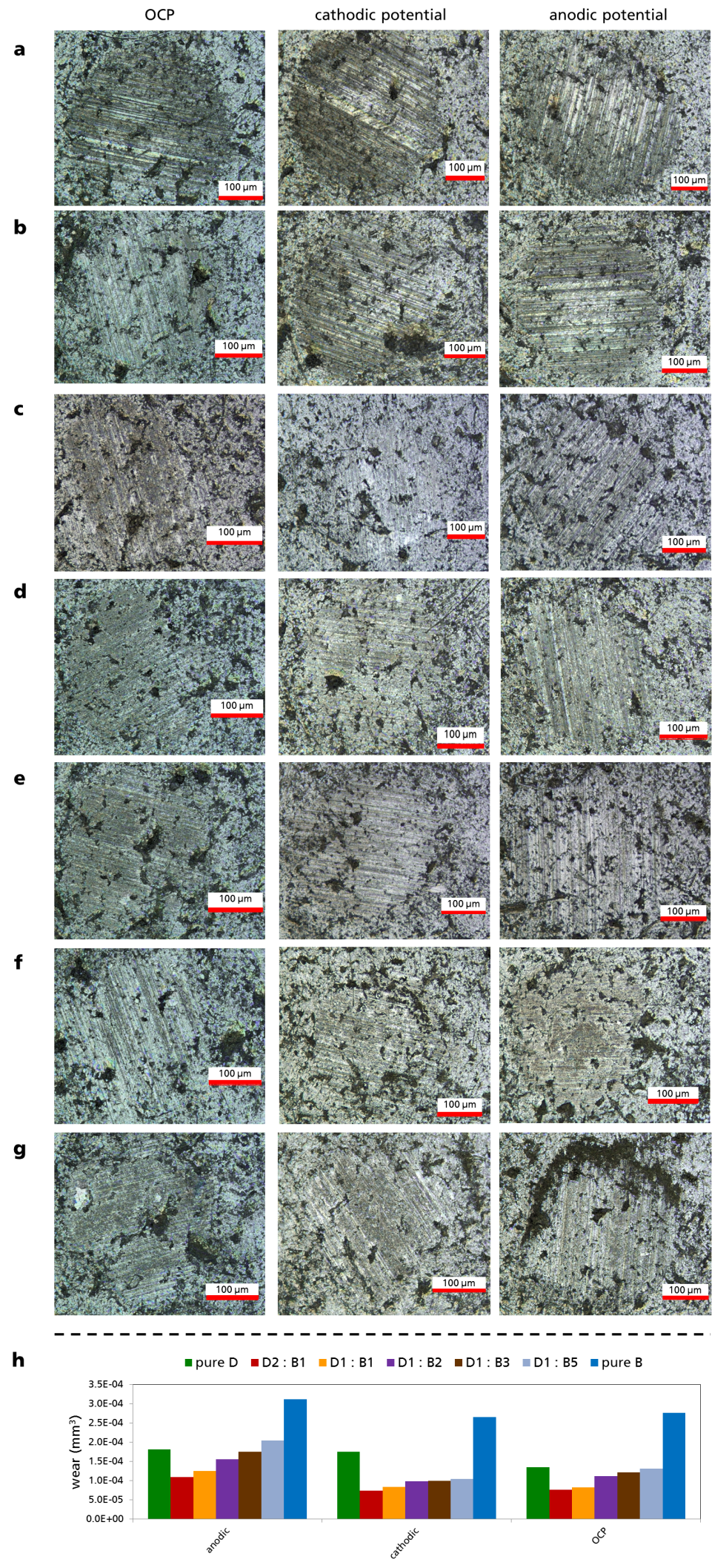


**Fig S3** Wear scar on pin analyzed with 3D-laser microscope (VK-9700K, Co. Keyence) after tribological test (50 N, 100 rpm, 0.05 m/s and room temperature) at cathodic and anodic polarization using as lubricant: **a** pure [P66614][DEHP] and **b** pure [P66614][BTA], as well as the mixtures of [P66614][DEHP] (D) and [P66614][BTA] (B) in a weight proportion of **c** 1:1 (D1:B1), **d** 2:1(D2:B1), **e** 1:2 (D1:B2), **f** 1:3 (D1:B3) and **g** 1:5 (D1:B5). **h** The wear volume on the three pins was measured by using the 3D-laser microscope. Also wear is affected by polarization and lower wear volume is reached at cathodic polarization. In addition, mixtures of the ILs show lower wear volume than the pure ILs.


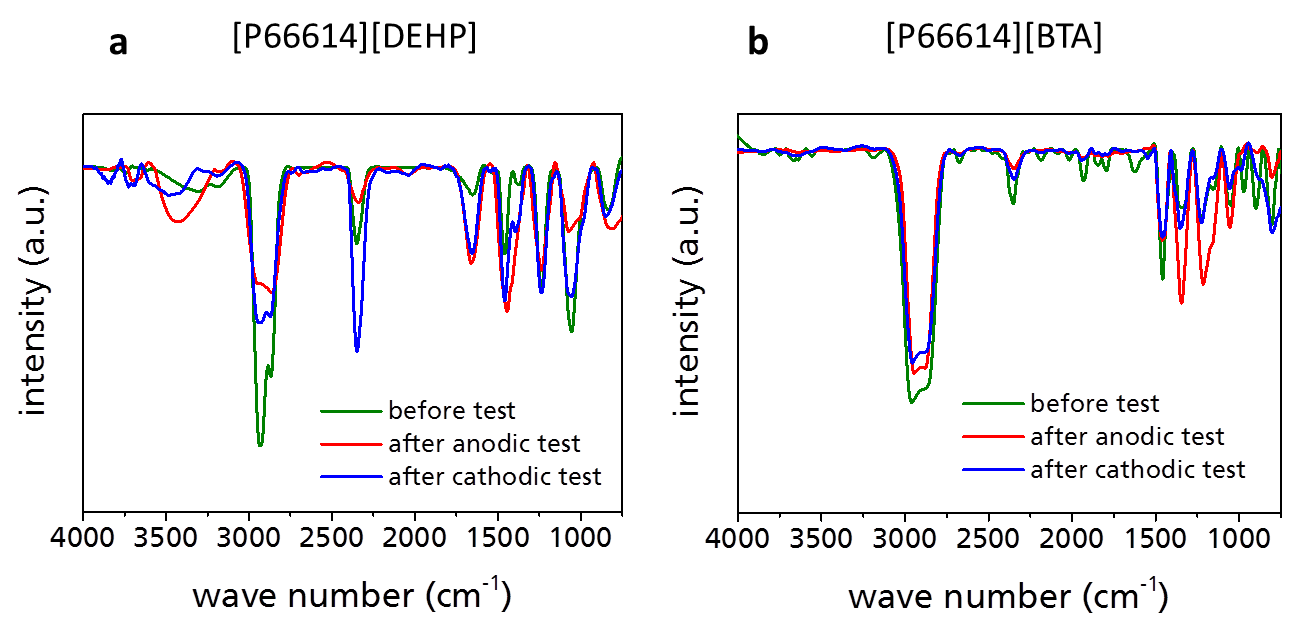


**Fig S4** Comparison of the IR-spectra (transmission, 4000cm^-1^-700cm^-1^) before (green line) and after a tribological test of **a** pure [P66614][DEHP] and **b** [P66614][BTA] consisting a running-in phase of 2h at OCP, an anodic (red line) or cathodic (blue line) polarization of 0.5h long and a running-out phase of 1.25h at OCP.


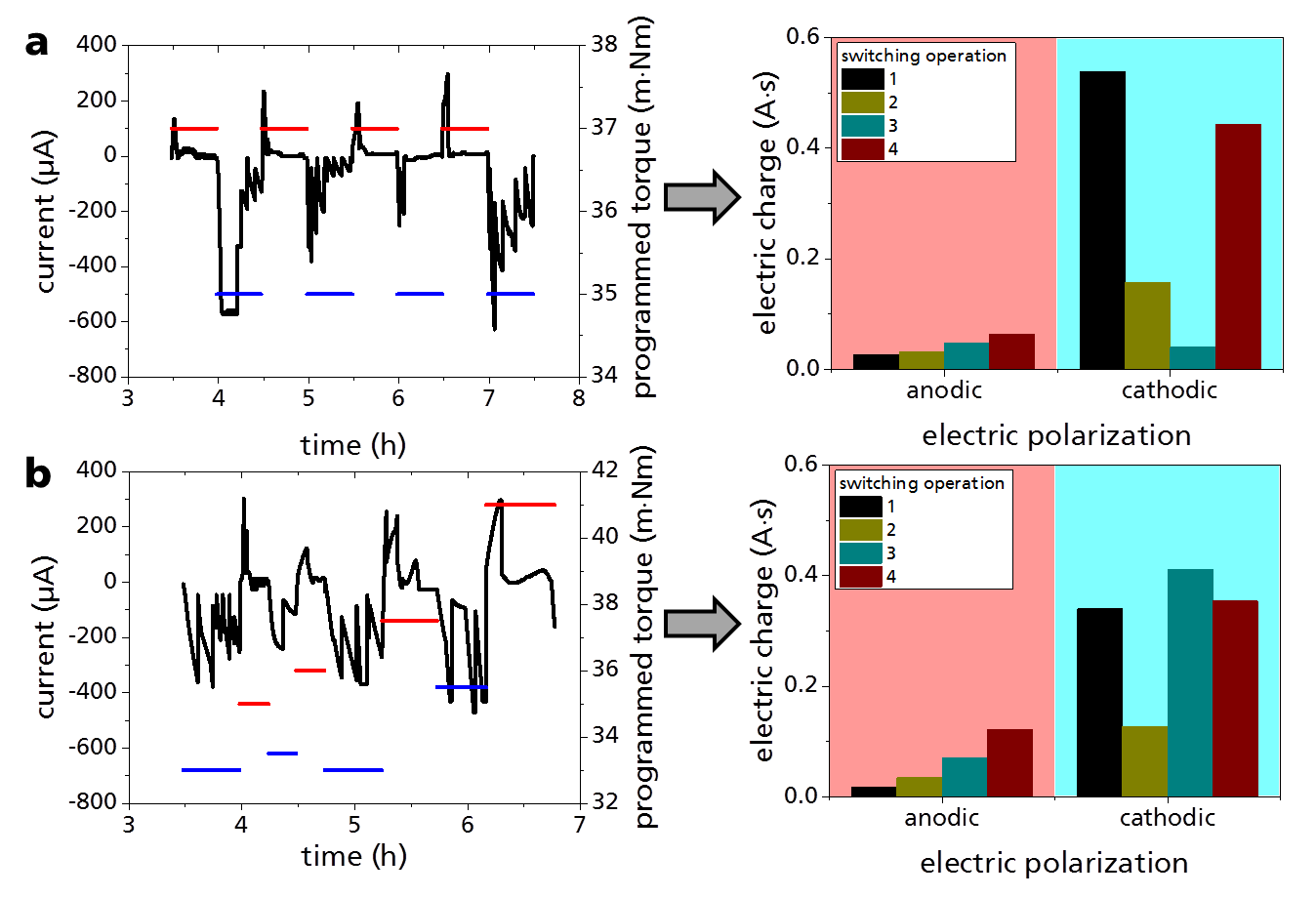


**Fig S5** Automatically regulated current with D1:B3 through the tribo-controller to reach the programmed torque values. Increase of torque is realized by anodic and reduction of torque by cathodic surface polarization. Current (left) and calculated electric charge quantity per programmed torque value (right) for multiple adjustment of **a** the same COFs 0.1180 and 0.1115 and **b** for multiple adjustment of different COFs.


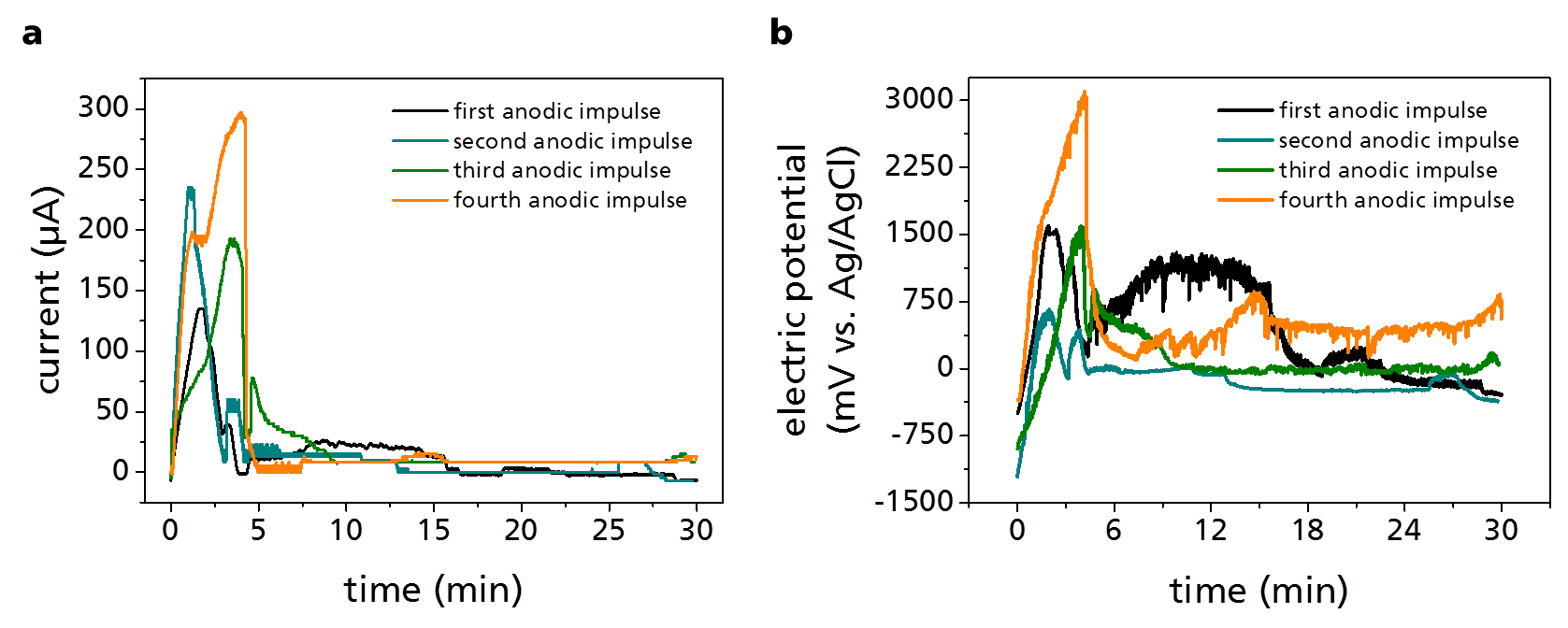


**Fig S6** Superposed anodic switching operations from figure S5a. Current impulse (**a**) and potential impulse (**b**) within the first 5 minutes is sufficient to cause a change in COF. After reaching the programmed COF the current decreases to almost zero.

**
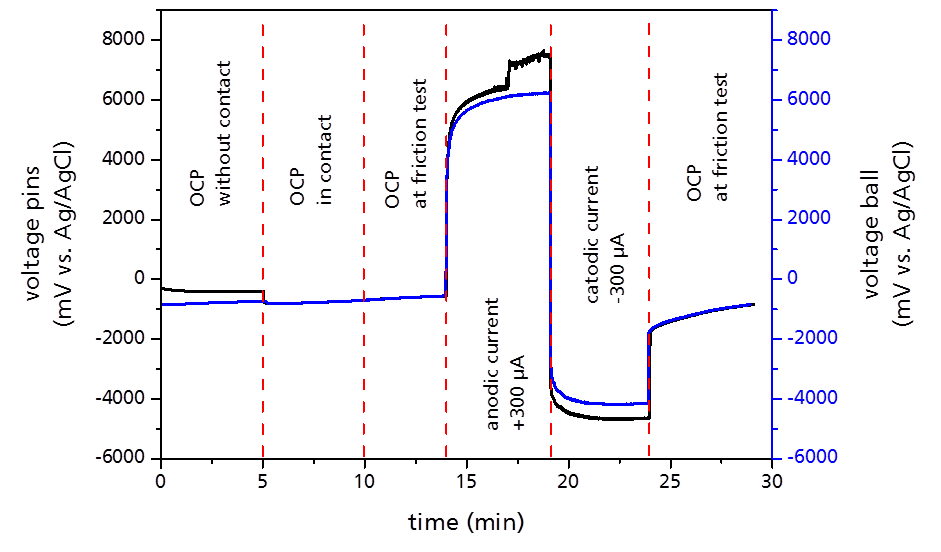
**

**Fig S7** Comparison of the voltage applied to the pins (black line) with the potentiostat (Wenking LPG03, Bank Electronic–Intelligent Controls GMBH) and the voltage resulting on the ball (blue line). At standstill the OCP was measured without contact between ball and pins (5 min) and with contact (5 min). The friction test was then started (50N, 100 rpm, mixture D1:B3) and after further 4 minutes an anodic (+300 µA) and a cathodic (-300 µA) potential were applied for 5 minutes. The last 5 minutes the test was run at OCP.

**
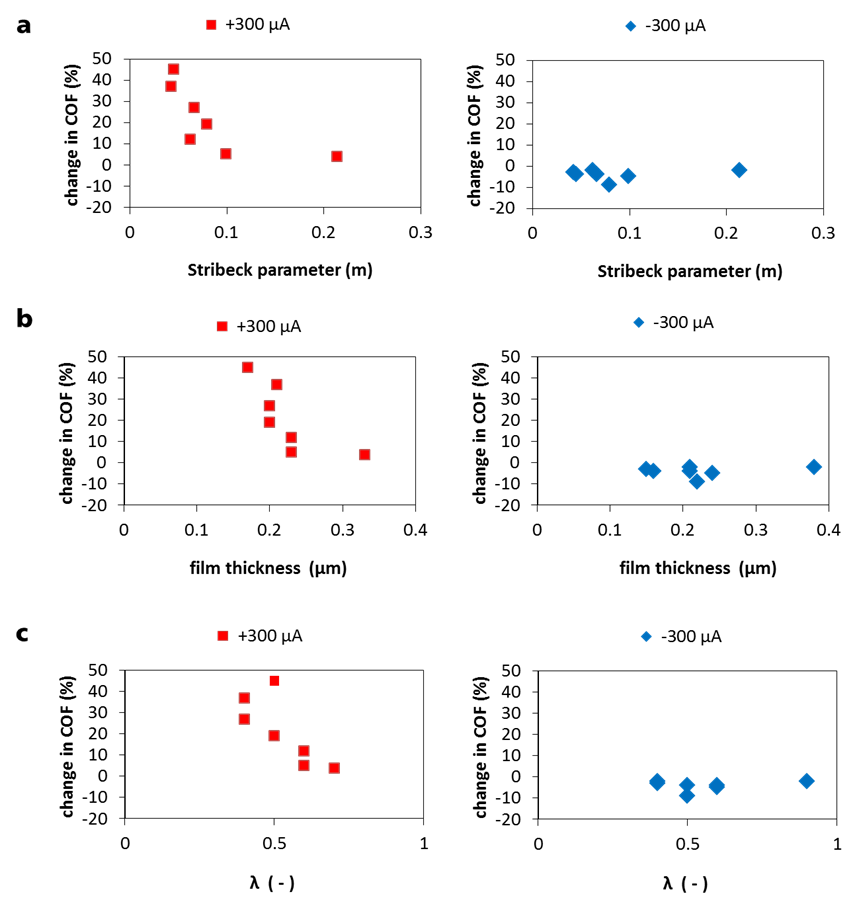
**

**Fig S8** **a** Correlation of the Stribeck parameter (viscosity∙velocity/contact pressure) and the change of COF. The viscosity of pure [P66614][DEHP] and pure [P66614][BTA], as well as the mixtures D1:B1, D2:B1, D1:B2, D1:B3 and D1:B5 was determined at 25 °C (Fig S15). The lubricant film thickness was calculated as a function of viscosity, resulting contact pressure (Fig S3) and speed of the rotating ball. **b** Correlation of change in COF with lubricant film thickness. The anodic values (left, red squares) result from experiments at +300 µA and 50 N. The cathodic values (right, blue diamonds) from experiments at -300 µA and 50 N. **c** Correlation of change of COF and lambda value, which is the quotient of the lubricant film thickness and the combined roughness of the two bodies.


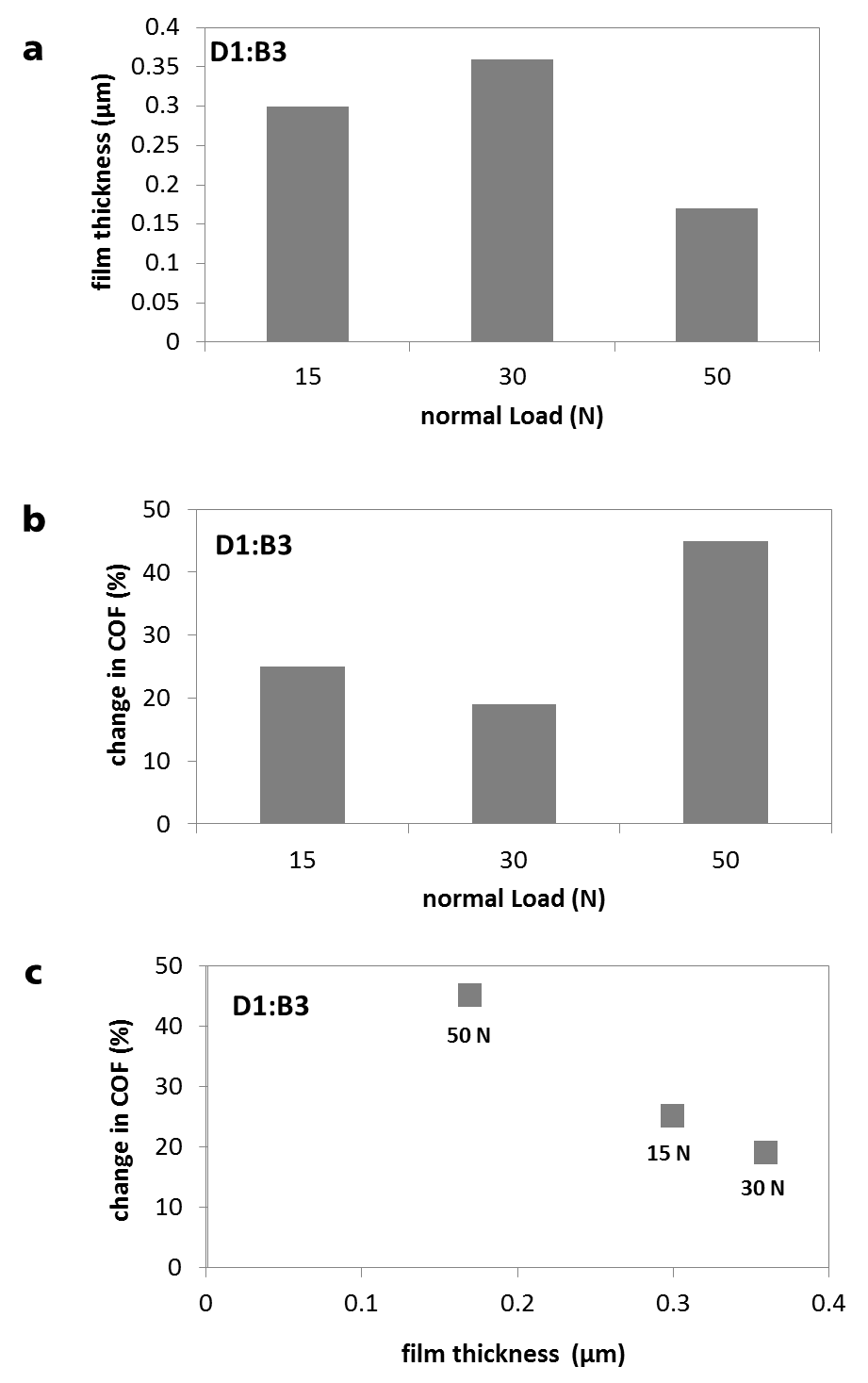


**Fig S9** Tribological tests using reduced normal load of 15 N and 30 N using mixture D1:B3 at an anodic current of +300 µA. **a** Resulting lubricant film thickness at different normal loads. Film thickness was calculated as a function of viscosity, resulting contact pressure and speed of the rotating ball. **b** Change of COF by application of anodic potential at different normal loads. **c** Change of COF depending on the lubricant film thickness. For comparison the values of the experiment with D1:B3 at 50 N and 300 µA were added.


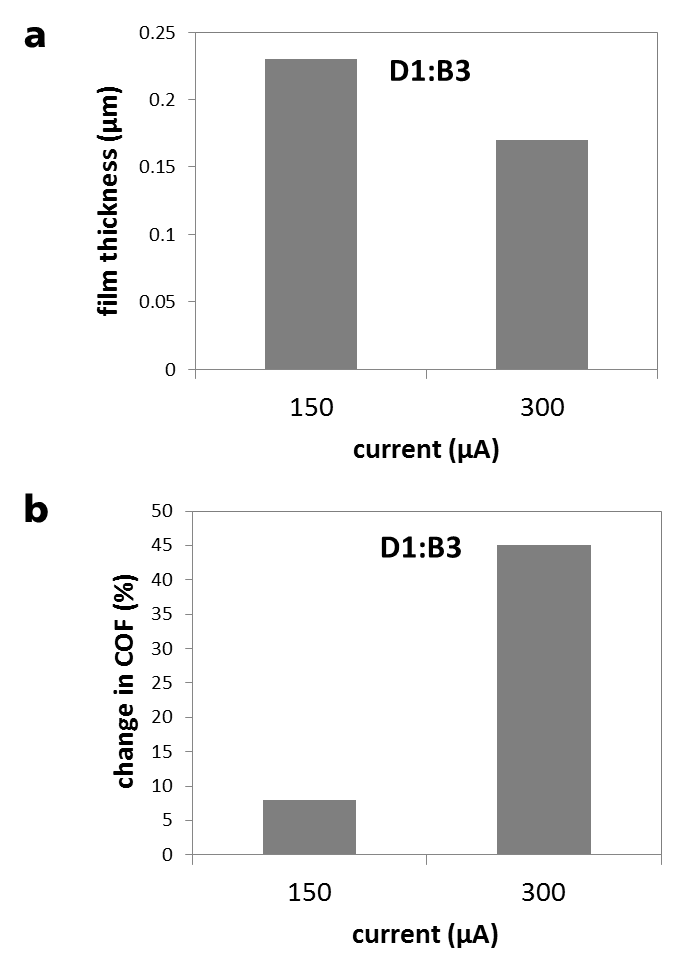


**Fig S10** Tribological tests using a lower anodic current as trigger of +150 µA using mixture D1:B3 and a normal load of 50 N. **a** Values of the film thickness at the experiments with 150 µA and 300 µA. Film thickness was calculated as a function of viscosity, resulting contact pressure and speed of the rotating ball. **b** Change of COF at 150 µA and 300 µA. For comparison the values of the experiment with D1:B3 at 50 N and 300 µA were added.


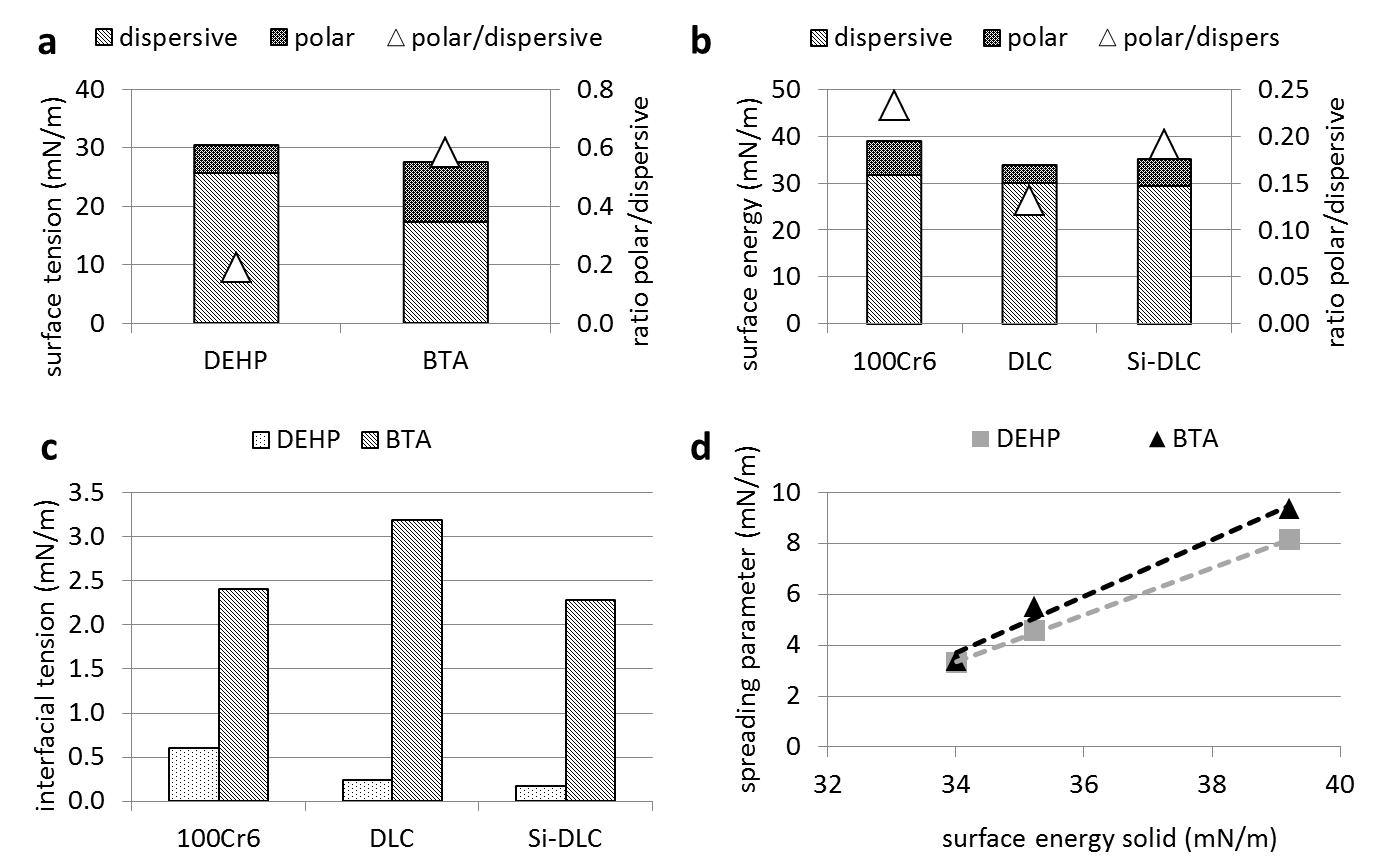


**Fig S11** Interfacial parameters were measured to analyze the adhesive strengths between liquid and solid in the frictional contact. Surface energies of the different solid materials and their dispersive (D) and polar (P) properties were measured using sessile drop method. In combination with the measured surface tension using pendant drop method the polar and dispersive components of the lubricants were calculated. According to Fowkes method, the interfacial tension is calculated based on the surface energy of the solid and the surface tension of the lubricant and their polar and dispersive components. Comparison of surface tension of the ionic liquids [P66614][DEHP] and [P66614][BTA] (**a**) and the surface energy of steel, DLC and Si-DLC (**b**); **c** Calculation of interfacial tension of the two ionic liquids with different surfaces; **d** Calculation of spreading parameter of the two ionic liquids with different surfaces. Based on these values the interaction of solid and lubricant can be estimated by the spreading parameter (SP) since a solid surface becomes completely wetted when SP > 0 in order to lower its surface energy and partial wetting occurs for SP < 0. Lower SP indicates more oleo-phobic behavior that means poorer, oil-wetting behavior.


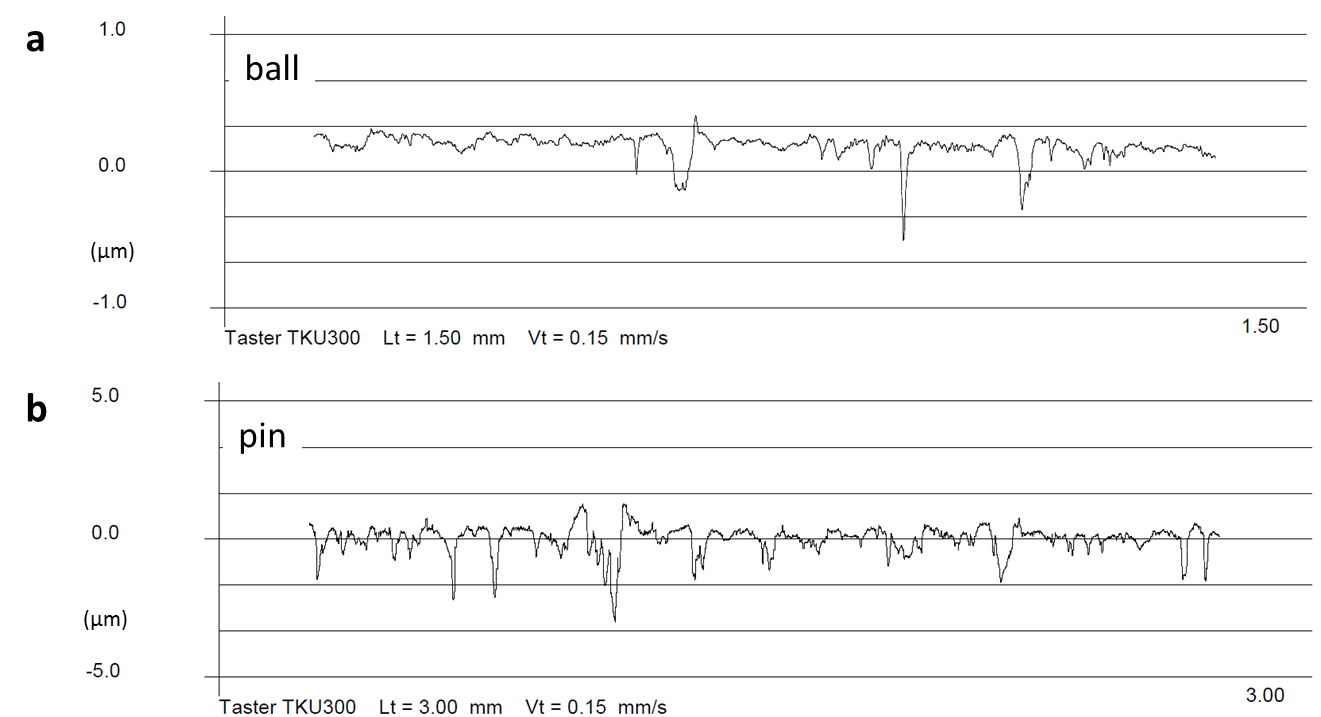


**Fig S12** Surface profile of 100Cr6 ball (**a**) and pin (**b**) measured using profilometer (Wave System T8000, Co. Hommelwerke). Resulting roughness values for ball (R_a_ = 0.26 ± 0.06 µm, R_z_ = 0.57 ± 0.14 µm) and pins (R_a_ = 0.32 ± 0.05 µm, R_z_ = 2.47 ± 0.29 µm).


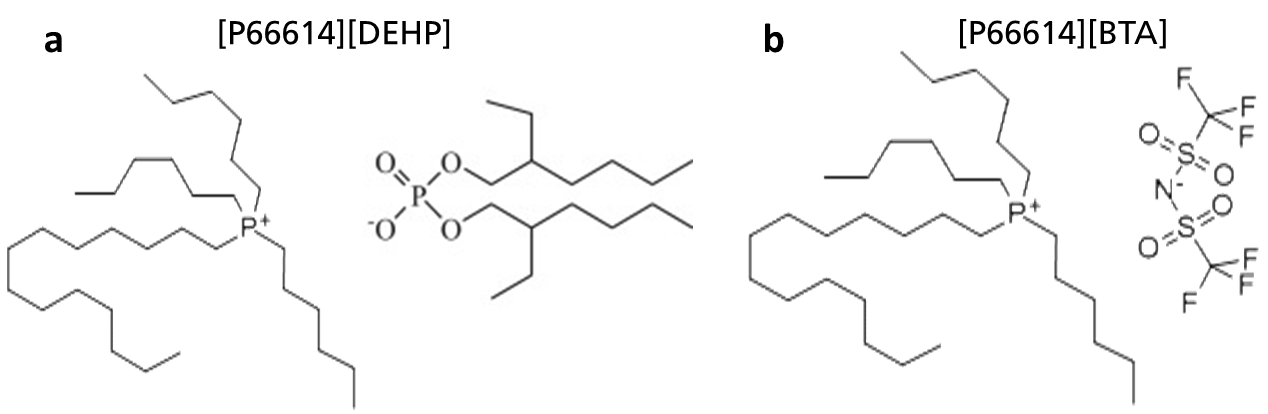


**Fig S13** Chemical structure of **a** Trihexyltetradecylphosphonium bis(2-ethyl-hexyl)phosphate [P66614][DEHP] (CS-0957, Co. Iolitec) and **b** Trihexyltetradecylphosphonium bis (trifluoromethylsulfonyl) imide [P66614][BTA] (IN-0021, Co. Iolitec).


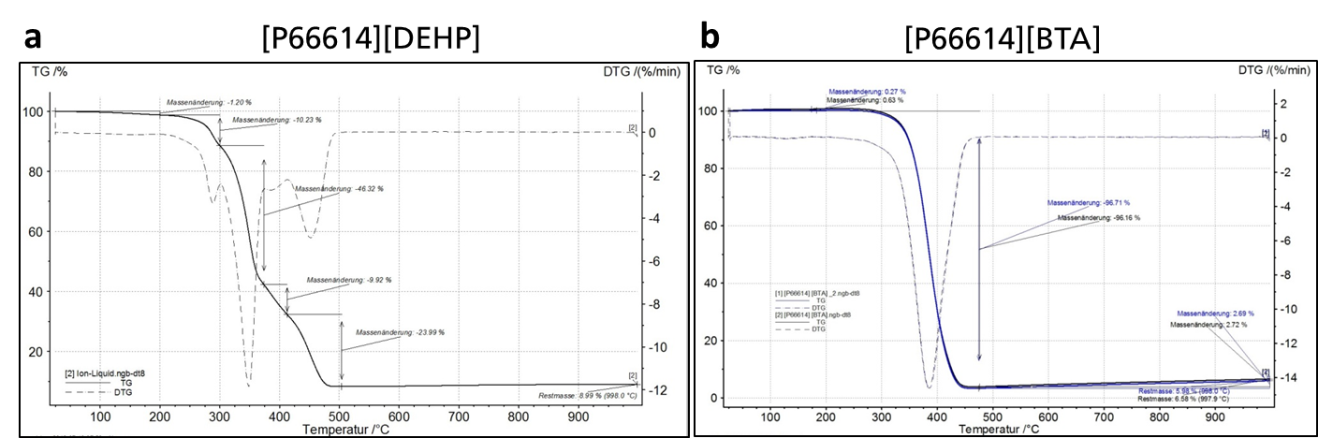


**Fig S14** Thermogravimetric analysis of **a** [P66614][DEHP] an **b** [P66614][BTA] to determine the decomposition temperature. **a** [P66614][DEHP]: Under N_2_ inert gas up to 200 °C a very small mass loss of approx. 1.2 % can be observed. The main decomposition of the sample takes place in several merging mass steps. Up to 300 °C the sample loses about 10% of its mass, up to 375 °C another 46 %, up to 410°C 9% and up to 500 °C 23 %. The decomposition is completed at about 500 °C; **b** [P66614][BTA]: Under N_2_ inert gas a very small mass loss of approx. 0.5 % can be observed up to 200 °C. The main decomposition of the sample is completed at 470 °C.


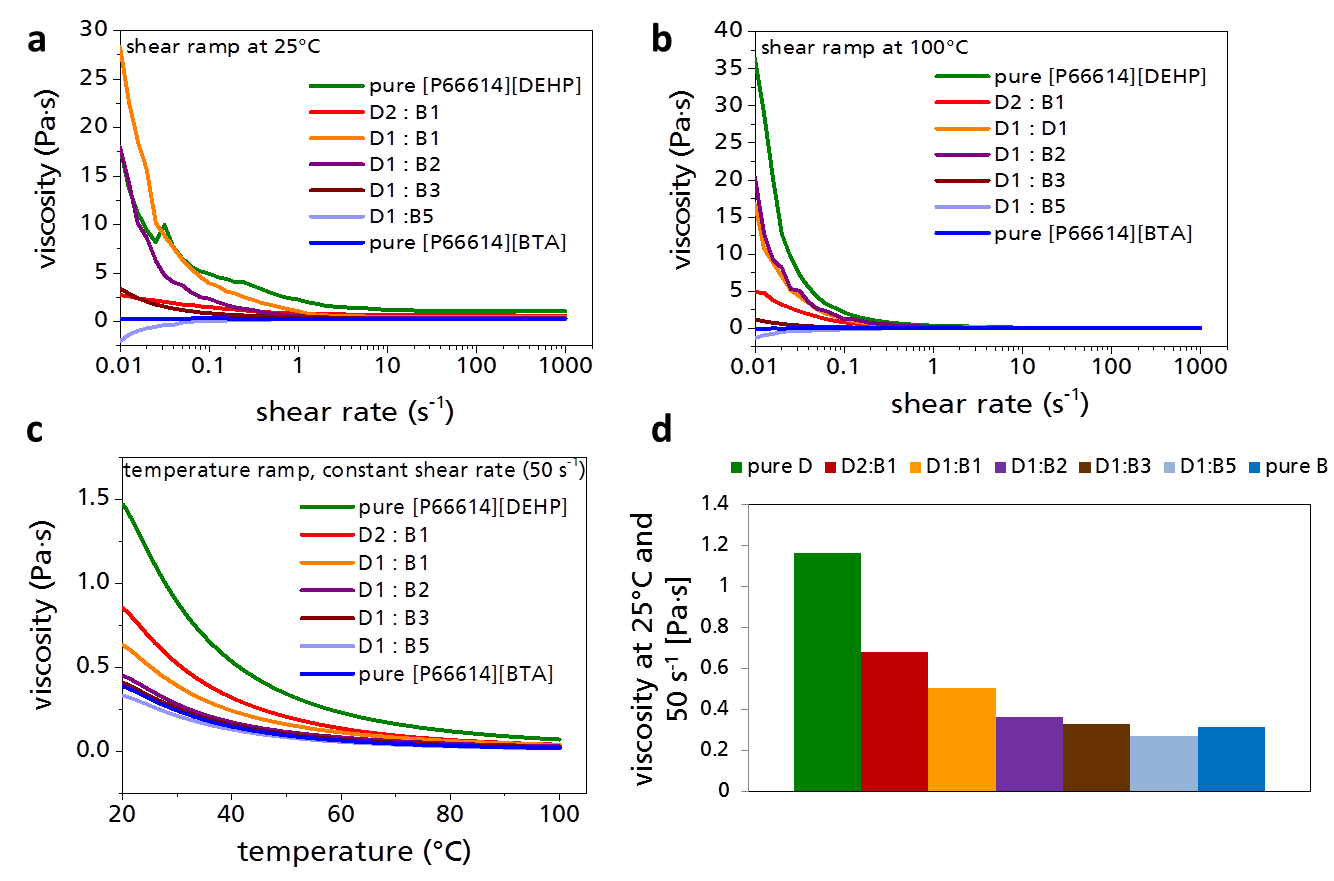


**Fig S15** Rheological analysis of pure [P66614][DEHP] and pure [P66614][BTA] as well as the mixtures of [P66614][DEHP] and [P66614][BTA] in a weight proportion of 2:1 (D2:B1), 1:1 (D1:B1), 1:2 (D1:B2), 1:3 (D1:B3) and 1:5 (D1:B5). Measurement of shear rate dependency of viscosity at **a** 25°°C and **b** 100 °C; **c** Influence of temperature on viscosity. **d** Comparison of viscosity values at 25 °C and shear rate of 50 s^-1^.


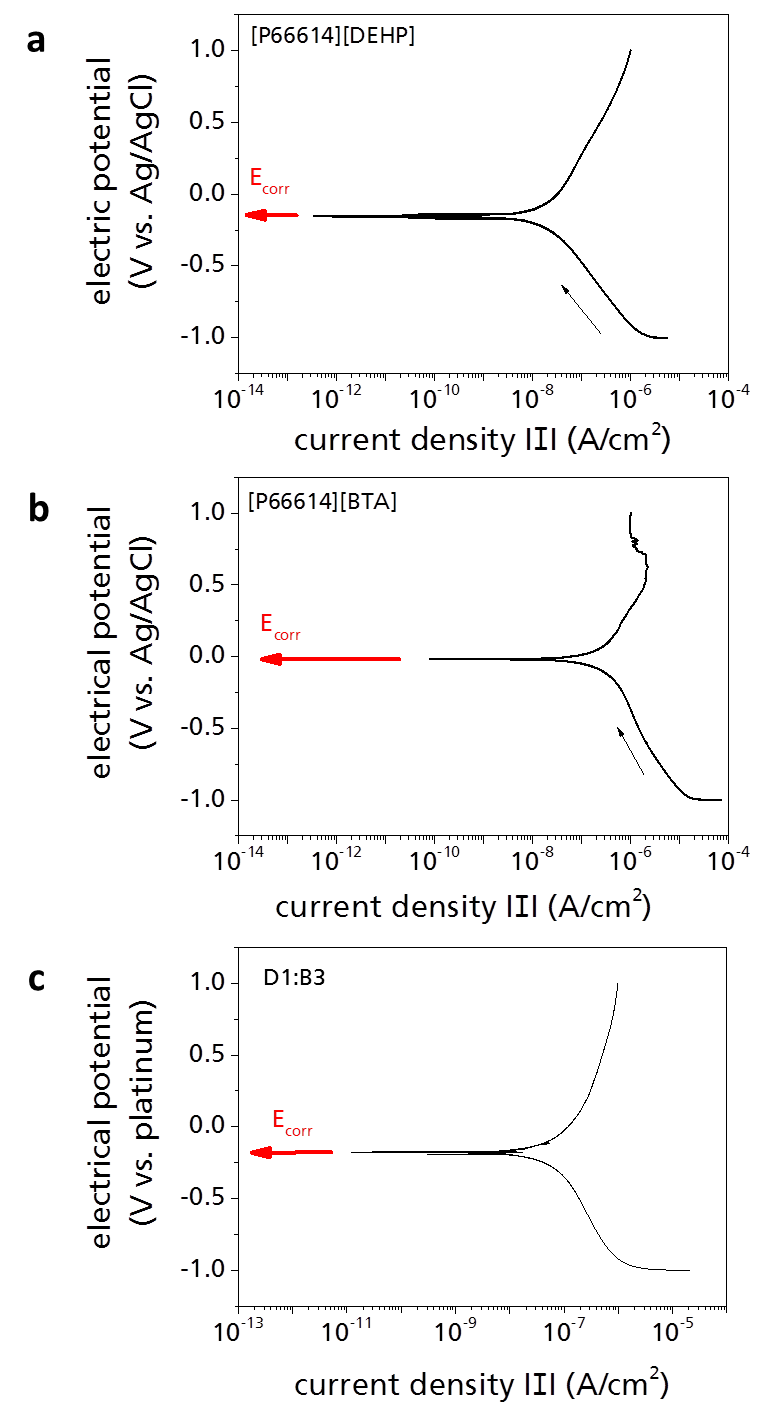


**Fig S16** Result of linear sweep voltammetry of **a** pure [P66614][DEHP], **b** [P66614][BTA] and **c** the mainly used mixture D1:B3 with 100Cr6 pins (scan rate: 0.1 mV/s, potential range: −1 to +1 V) using a potentiostat (Parstat 4000, Ametek).

**
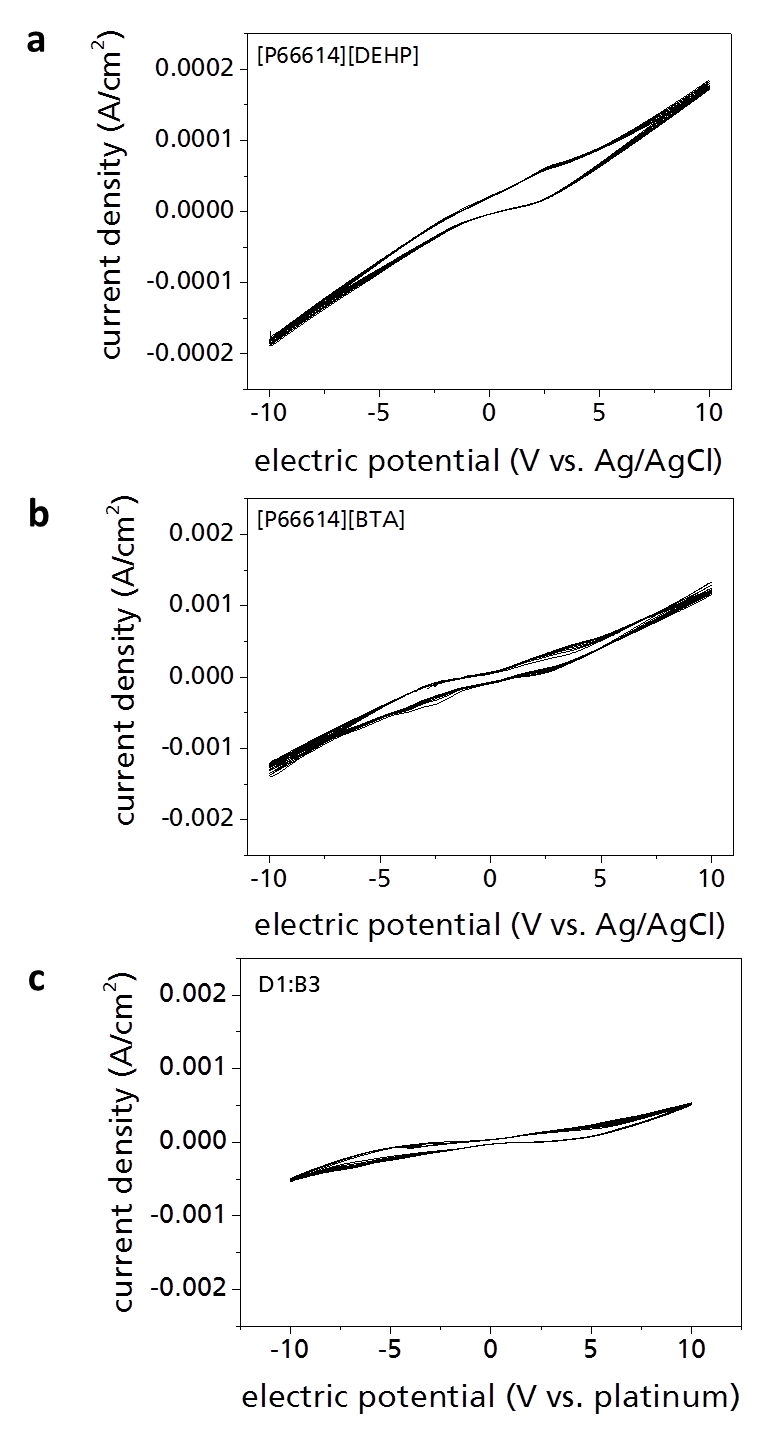
**

**Fig S17** Result of cyclic voltammetry (CV, scan rate: 100 mV/s, potential range: −10 to +10 V, 10 cycles) of **a** pure [P66614][DEHP], **b** pure [P66614][BTA] and **c** the mainly used mixture D1:B3 using a potentiostat (Parstat 4000, Ametek).

| IL-mixtures  normal force | electric charge | R_a_ after test | viscosity | wear area | resulting contact pressure | film thickness | lambda |
| --- | --- | --- | --- | --- | --- | --- | --- |
|  |  | (µm) | (Pa·s) | (m^2^) | (MPa) | (µm) | (-) |
| pure D | OCP | 0.27 | 1.1610 | 6.82E-08 | 346 | 0.31 | 0.8 |
| 50 N | +300 µA | 0.37 |  | 7.23E-08 | 326 | 0.33 | 0.7 |
|  | -300 µA | 0.32 |  | 8.66E-08 | 272 | 0.38 | 0.9 |
| pure B | OCP | 0.22 | 0.3114 | 7.48E-08 | 315 | 0.17 | 0.5 |
| 50 N | +300 µA | 0.25 |  | 1.08E-07 | 219 | 0.23 | 0.6 |
|  | -300 µA | 0.39 |  | 9.41E-08 | 251 | 0.21 | 0.4 |
| D2 : B1 | OCP | 0.24 | 0.6775 | 6.66E-08 | 354 | 0.24 | 0.7 |
| 50 N | +300 µA | 0.33 |  | 6.50E-08 | 362 | 0.23 | 0.6 |
|  | -300 µA | 0.29 |  | 6.90E-08 | 342 | 0.24 | 0.6 |
| D1 : B1 | OCP | 0.21 | 0.5047 | 6.47E-08 | 364 | 0.20 | 0.6 |
| 50 N | +300 µA | 0.26 |  | 6.58E-08 | 358 | 0.20 | 0.5 |
|  | -300 µA | 0.35 |  | 7.37E-08 | 320 | 0.22 | 0.5 |
| D1 : B2 | OCP | 0.37 | 0.3612 | 6.95E-08 | 339 | 0.18 | 0.4 |
| 50 N | +300 µA | 0.40 |  | 8.27E-08 | 285 | 0.20 | 0.4 |
|  | -300 µA | 0.23 |  | 8.60E-08 | 274 | 0.21 | 0.6 |
| D1 : B3 | OCP | 0.24 | 0.3280 | 5.93E-08 | 397 | 0.15 | 0.4 |
| 50 N | +300 µA | 0.25 |  | 7.03E-08 | 335 | 0.17 | 0.5 |
|  | -300 µA | 0.20 |  | 6.51E-08 | 362 | 0.16 | 0.5 |
| D1 : B5 | OCP | 0.37 | 0.3436 | 8.42E-08 | 280 | 0.20 | 0.4 |
| 50 N | +300 µA | 0.40 |  | 8.87E-08 | 266 | 0.21 | 0.4 |
|  | -300 µA | 0.24 |  | 5.76E-08 | 409 | 0.15 | 0.4 |
| D1 : B3 | +300 µA | 0.12 | 0.3280 | 6.59E-08 | 107 | 0.30 | 1.0 |
| 15 N |  |  |  |  |  |  |  |
| D1 : B3 | +300 µA | 0.14 | 0.3280 | 8.56E-08 | 83 | 0.36 | 1.2 |
| 30 N |  |  |  |  |  |  |  |
| D1 : B3 | +150 µA | 0.24 | 0.3280 | 1.07E-07 | 220 | 0.23 | 0.8 |
| 50 N |  |  |  |  |  |  |  |

**Table S1.** Overview of resulting surface roughness on pin after the tests (Fig S1), measured viscosity, resulting contact pressure due to wear area (Fig S3) and calculated lubrication film thickness and lambda values of pure [P66614][DEHP] (D) and pure [P66614][BTA] (B) as well as the mixtures of [P66614][DEHP] and [P66614][BTA] in a weight proportion of 2:1 (D2:B1), 1:1 (D1:B1), 1:2 (D1:B2), 1:3 (D1:B3), 1:5 (D1:B5). The results with D1:B3 at lower normal force (15 N and 30 N) and at lower current (+150 µA) are included.

| IL | E*_Corr_* | i*_Corr_* | C*_R_* |
| --- | --- | --- | --- |
|  | (mV vs. Ag/AgCl) | (A/cm²) | (mm/y) |
| pure D | −149 | 1.2E-8 | 1.4E-4 |
| pure B | −14 | 2.4E-7 | 2.7E-3 |

**Table S2.** Results of electrochemical measurements of pure [P66614][DEHP] (D) and pure [P66614][BTA] (B). Using the measurements of Fig S13 corrosion potential (E*_Corr_*), corrosion current density (i*_Corr_*) and corrosion rate (C*_R_*) was calculated.
